# A prospective randomized, double-blind, placebo-controlled, dose-response relationship study to investigate efficacy of fructo-oligosaccharides (FOS) on human gut microflora

**DOI:** 10.1038/s41598-019-41837-3

**Published:** 2019-04-02

**Authors:** Disha Tandon, Mohammed Monzoorul Haque, Manoj Gote, Manish Jain, Anirban Bhaduri, Ashok Kumar Dubey, Sharmila S. Mande

**Affiliations:** 1 Bio-Sciences R&D Division, TCS Research, Tata Consultancy Services Ltd., 54-B, Hadapsar Industrial Estate, Pune, 411 013 Maharashtra India; 2 Tata Chemicals Ltd. Innovation Centre, Survey Number 315, Hissa Number 1-14, Ambedveth, Mulshi, Pune, 412 111 Maharashtra India

## Abstract

Fructo-oligosaccharides (FOS), a prebiotic supplement, is known for its Bifidogenic capabilities. However, aspects such as effect of variable quantities of FOS intake on gut microbiota, and temporal dynamics of gut microbiota (transitioning through basal, dosage, and follow-up phases) has not been studied in detail. This study investigated these aspects through a randomized, double-blind, placebo-controlled, dose-response relationship study. The study involved 80 participants being administered FOS at three dose levels (2.5, 5, and 10 g/day) or placebo (Maltodextrin 10 g/day) during dosage phase. Microbial DNA extracted from fecal samples collected at 9 intervening time-points was sequenced and analysed. Results indicate that FOS consumption increased the relative abundance of OTUs belonging to *Bifidobacterium* and *Lactobacillus*. Interestingly, higher FOS dosage appears to promote, in contrast to Maltodextrin, the selective proliferation of OTUs belonging to *Lactobacillus*. While consumption of prebiotics increased bacterial diversity, withdrawal led to its reduction. Apart from probiotic bacteria, a significant change was also observed in certain butyrate-producing microbes like *Faecalibacterium*, *Ruminococcus* and *Oscillospira*. The positive impact of FOS on butyrate-producing bacteria and FOS-mediated increased bacterial diversity reinforces the role of prebiotics in conferring beneficial functions to the host.

## Introduction

The human gut harbours one of the most densely populated microbial communities on earth^1^. Billions of bacteria coat the mucus lining of the gastrointestinal tract. Estimates indicate that bacterial density in the distal portions of the gut (i.e. colon) reaches to around ~10^11^ to 10^12^ colony forming units (CFUs) per gram of luminal content^2,3^. Besides aiding in the process of digestion, gut bacteria have been shown to play a crucial role in maintaining immune and metabolic homoeostasis^4^.

The structure and functional potential of gut microbial communities is defined/modulated by a plethora of factors which may be host-defined (e.g. genetics, age, weight, ethnicity, level of physical/mental activity, stress, etc.,) or environmental (e.g. diet, external infections/injury, medications, living conditions, etc.). Several studies have attempted to decipher the nature of associations between diet, the composition/metabolic potential of the human gut microbiome, and human health^5,6^. It is becoming increasingly clear that a healthy gut (microbiome) confers (and contributes to) good health and overall well-being. Besides trying to understand the consequences of (evolving) dietary patterns on gut microbiota (and eventually individual health status), recently there has been a renewed interest in re-visiting methods/compositions that can be employed for restoring or maintaining the gut microbiota in a favourable healthy state^7–9^. Nutraceutical food supplements or specific dietary ingredients (e.g. fiber, probiotics, prebiotics, etc.) are now being investigated from the perspective of their impact on health via their influence on gastrointestinal microbiota.

Probiotic supplementation has been demonstrated as an effective method of (re-)introducing healthy bacteria back into the gut microbiome^10^. Emerging research however suggests that selective modulation of the composition and function of host gut microbiota through consumption of non-digestible food ingredients i.e. prebiotic supplements is an equally (if not more) effective nutritional strategy for maintaining/restoring host gut health (and thereby general well-being)^10–23^. In contrast to probiotics, prebiotic supplements act as fodder for colonic bacteria (already present within the gut ecosystem) which play a major role in metabolism of complex carbohydrates and plant polysaccharides (which human enzymes are unable to completely digest). The latter metabolism generates (as primary end products) SCFAs i.e. short-chain fatty acids, namely, acetate, propionate, and butyrate. Besides their role in various host-signalling pathways, SCFAs are known for their favourable modulatory effects on host immunity, gastrointestinal epithelial cell integrity, glucose homoeostasis, lipid metabolism, regulation of appetite and, immune function^24,25^. By serving as a predominant energy substrate for colonocytes and enterocytes, butyrate is a key metabolite affecting gut health. More importantly, prebiotic dietary supplements, by promoting/enabling cross-utilization of by-products between bacterial community members, help in (indirectly) stimulating the growth of other microbes thereby increasing bacterial diversity. This sort of indirect modulation (of gut microbial diversity) is known to have favourable metabolic consequences that cannot be achieved by probiotic supplements. Method-wise, the latter supplements (in contrast) are biased towards artificially populating the gut microbial ecosystem with a few select microbial isolates.

In this work, we conducted a single-centre, randomized, double-blind, placebo-controlled, dose-response relationship study to investigate the efficacy of Fructo-oligosaccharides (FOS) on human gut microflora. Inulin, FOS, and GOS (Galacto-oligosaccharides) have been amongst the most researched prebiotic supplements in recent time^26–31^. The primary objective of the study was to specifically evaluate the Bifidogenic properties of FOS (manufactured by Tata Chemicals Limited under the brand name Fossence^TM^) administered to healthy, adult, consenting human participants (n = 80) at three different dose levels viz., 2.5 g/d, 5.0 g/d and 10 g/d. A second objective was to measure the effect of FOS on random blood sugar, calcium, and triglycerides in healthy, adult, human participants. In addition to evaluating the effect of FOS (at different dosage levels) on fermentative microbes (*Bifidobacterium*, *Lactobacillus*), the scope of analysis was extended to study the effects of the administered FOS supplement on groups of microbes that can perform different functions, namely, butyrate-producing microbes, fibre-digesting microbes, etc. Following the precedent of earlier studies^17,32,33^, an additional placebo arm was included for comparison. This arm included a subset of the recruited participants who were administered 10.0 g of a non-prebiotic carbohydrate i.e. Maltodextrin (brand-name Glucidex DE12 procured from Roquette as placebo) during the dosage phase of the study. It may be noted that the degree of polymerization of the FOS supplement used in the present study is 3–5, and this molecule is not branched.

## Methods

This study was designed according to the CONSORT 2010. Study protocol was approved by the Anveshhan Independent Ethics Committee (Ahmedabad, Gujarat, India) associated with the study centre. The study (Clinical Trial Number: CTRI/2019/01/016937; Registered on: 07/01/2019; www.ctri.nic.in) was conducted (at Veeda Clinical Research, Ahmedabad, India) as per the pertinent requirements of the ICMR guidelines for Biomedical Research on Human Subjects, Good Clinical Practices for Clinical Research in India (amended version - 2005, Schedule Y, CDSCO guideline, ICH (Step 5) Guidance on Good Clinical Practice). The protocol was carried out in accordance with the approved guidelines, and was in agreement with Declaration of Helsinki principles (Brazil, October 2013). Prior to initiation of the study, willing volunteers were educated on various aspects of the study, its objectives and the sampling procedures involved. Necessary written consents were obtained from willing volunteers using relevant consent documents. All participants provided fully-informed, written consent. A sample template of the consent form which was used in the present study is provided as Supplementary File S1. Willing volunteers (n = 80) who were considered as having normal health (as determined by personal medical history, clinical and laboratory examinations) were recruited at Veeda Clinical Research Centre as per the following inclusion/exclusion criteria.

### Inclusion criteria

1. Age between 18 and 55 years (both inclusive).
2. Minimum 45 kg weight; BMI range between 18.50 to 30.00 kg/m^2^.
3. Participants having clinically accepted 12-lead electrocardiogram & chest X-Ray (PA view).
4. Participants having negative urine-screen for drugs of abuse (including amphetamines, barbiturates, benzodiazepines, marijuana, cocaine, and morphine).
5. Participants having negative alcohol breathe test.
6. Negative Urine Pregnancy test at screening on admission day 01 of basal phase (for female participants).

### Exclusion criteria

1. Hypersensitivity to Fructo-oligosaccharides or Maltodextrin or related class of drugs or any of its excipients or to heparin.
2. History or presence of significant cardiovascular, pulmonary, hepatic, renal, gastrointestinal, endocrine, immunological, dermatological, neurological or psychiatric disease or disorder.
3. Any treatment which could bring about induction or inhibition of hepatic microsomal enzyme system within 1 month prior to day 01 of basal phase.
4. History or presence of significant alcoholism, drug abuse, smoking (more than 10 cigarettes/beedis per day), history of major illness within past 3 months or cancer.
5. Use of any prescribed medication during last one month or OTC medication during last two weeks prior to day 01 of basal phase.
6. Volunteer who have donated blood (1 unit) within 90 days prior to the first dose of the study drug or have blood loss, excluding volume drawn at screening (more than 100 ml within 30 days; more than 200 ml within 60 days) prior to day 01 of basal phases of the study or have received a known investigational drug within five elimination half-life of the administered drug prior to the first dose of the study drug.
7. Consumption of grapefruit juice, xanthine-containing products, tobacco containing products or alcohol within 48 hours prior to day 01 of basal phase.
8. Positive screening test result for any one or more: HIV, Hepatitis B, and Hepatitis C.
9. History or presence of significant easy bruising, bleeding, or recent trauma.
10. Participants who have been on an abnormal diet (for whatever reason) during the four weeks preceding the study.
11. Female participants who are currently breast feeding.

### Demography and other baseline characteristics

All 80 study participants were from Ahmedabad and were from a lower socio-economic background. Supplementary Table S1 provides Metadata information (age, sex, dietary preferences, and other vital stats collected at all nine time-points) corresponding to the participants. The normal diet of participants comprised of locally available fruits and vegetables, wheat, millet, sorghum, dairy-products, sprouts, leafy vegetables, rice, and pulses. Meat/fish consumption in non-vegetarian participants was occasional and quantity was limited.

### Study Design

The study duration (210 days) was divided into three phases comprising a total of nine time-points (involving sample collection).

1. Basal Phase - Two time-points (Day 01 and Day 60)
2. Dosage Phase - Four time-points (Days 75, 90, 120, and 150)
3. Follow-up Phase - Three time-points (Days 165, 180, and 210)

**Figure 1:**
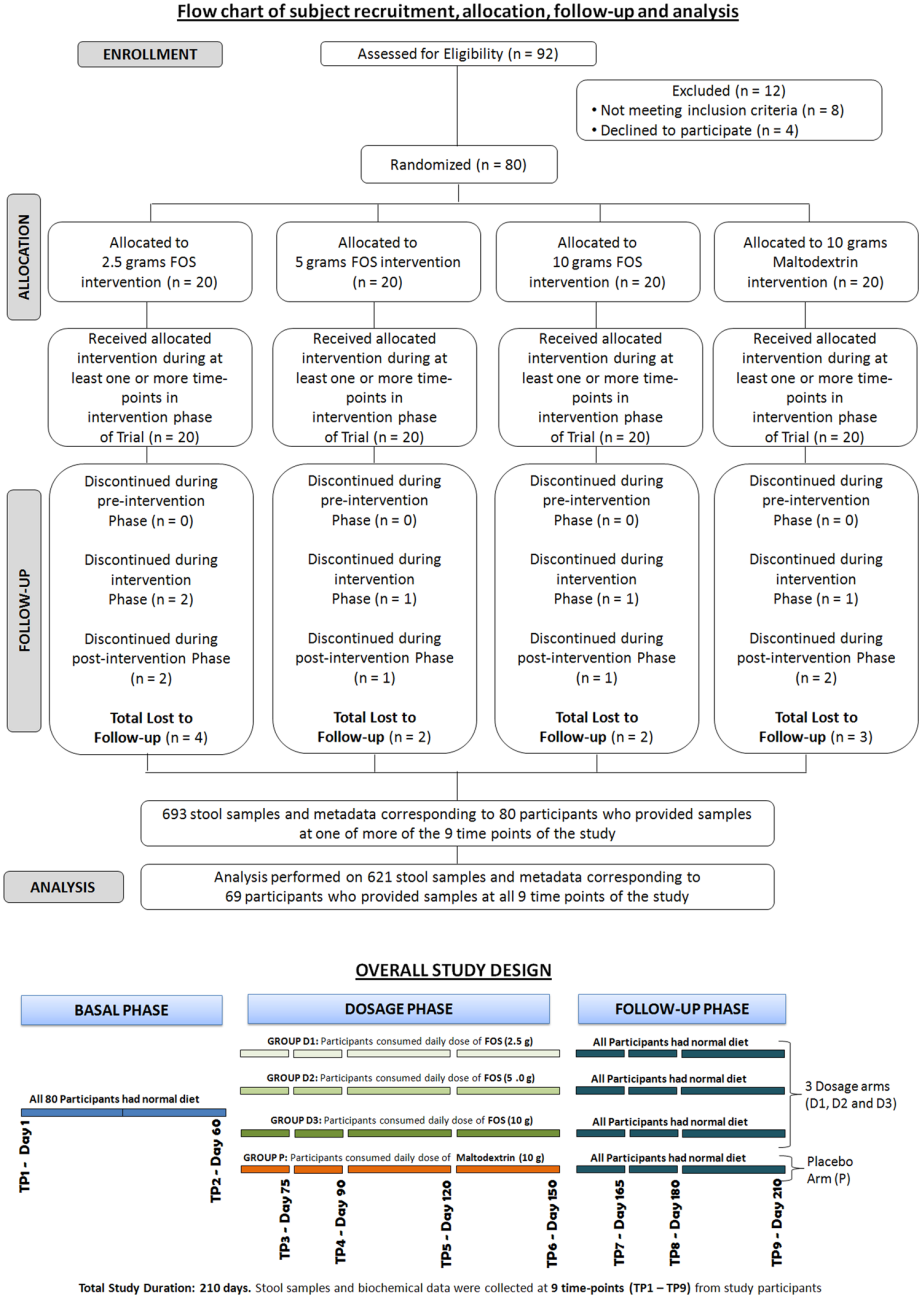
Schematic diagram depicting overall study design. The study corresponded to randomized, double-blind, four-arm parallel-group, placebo-controlled and dose-response relationship. Post-study safety assessment (Haemoglobin, Total Count, Differential Count, Platelet count, and biochemical parameters –SGOT, SGPT, Bilirubin, Creatinine, and Urea) was also performed at the end of study (Day 210).

As depicted in Fig. 1, through a randomization procedure, study participants were assigned to one of four groups, viz., P, D1, D2, and D3. Randomization was carried out by a trained investigator at Veeda Clinical Research Centre, Ahmedabad using SAS (SAS® Institute Inc., USA) Version 9.2 and was done in blocks using PROC PLAN such that the design was balanced. The order of receiving the FOS (test) and placebo for each subject in the study was determined according to randomization schedule. The randomization code and investigational product dispensing record were kept in the pharmacy under controlled access till the un-blinding procedure and after that they were kept in study file for archiving. No particular procedure was followed for determining the sample size.

During the Dosage Phase, participants in group P were instructed to consume (from day 60 to day 150) a daily dose of maltodextrin (10.0 g). Participants were instructed to dissolve each dose in 240 ml of drinking water at ambient temperature and consumed the same daily after dinner. In a similar manner, participants in groups D1, D2, and D3 consumed 2.5 g, 5.0 g and 10.0 g of FOS, respectively. The study design however did not include negative and positive controls.

At all nine time-points, all participants were instructed to visit the clinical facility (preferably in the morning) for providing stool and blood samples. Schedule deviations of a maximum of ±1 days were allowed. On Day 01 of the study, each participant underwent a physical, systemic, and clinical examination in which vital signs (viz. sitting blood pressure, oral body temperature, radial pulse rate, and respiratory rate) were assessed and recorded. Participants were then asked to provide stool and blood samples. Although, participants were instructed to consume their normal diet and water during the course of study, they were asked to limit (and preferably refrain) consumption of fermented dairy products and foods such as onions, wheat, artichokes, etc., which are known to contain high levels of non-digestible oligosaccharides. For safety-monitoring, vital signs and observations of clinical examination for all participants were recorded as per protocol. Post-study safety assessment (haemoglobin, total count, differential count, platelet count and biochemical parameters –SGOT, SGPT, Bilirubin, Creatinine, and Urea) was done at the end of study (Day 210).

In total, from each participant, nine blood and stool samples were planned to be collected during the study on day 1, 60, 75, 90, 120, 150, 165, 180 and 210 over a period of seven months. Stool sample collection was performed at the Veeda collection center. Immediately after obtaining a sample from a study participant, a trained technician (following the kit protocol) transferred the sample into a pre-labelled OMNIgene^®^-GUT (OMR200) stool collection tube. These tubes were procured from DNA Genotek, Canada. Samples collected in the OMNIgene^®^ GUT stool collection kit were stored at ~20 °C. The mentioned temperature was within the storage range as per the kit manufacturer’s instructions. The temperature of ~20 °C was maintained until further processing of the samples at the central sequencing facility. Transfer of the stool samples to the sequencing site (Genotek) was done as per defined recommendations and necessary precautions. Blood samples were collected in pre-labelled vacutainers and were transported to Supratech Micropath Laboratory & Research Institute, Ahmedabad, India for analysis. In all groups of participants viz., P, D1, D2, and D3, while the blood samples collected at each time point were analyzed for measuring random blood sugar, calcium, and triglyceride levels, stool samples were subjected to microbial DNA extraction and subsequent 16S rRNA amplicon sequencing for obtaining microbiome 16S sequence data corresponding to each of the collected samples. It may be noted that this study is a continuation of an earlier published study^34^ and comprises of the same participant cohort. The microbiome characteristics of stool samples collected during the basal phase (in the mentioned study) are described in detail in Tandon *et al*.^34^.

### Genomic DNA extraction

DNA from each stool sample was extracted using Qiagen DNeasy Blood & Tissue Kit. Nucleic acid concentration and purity was estimated spectro-photometrically. Library preparation was performed at Genotypic Technology’s Genomics facility. 4 ng of nano-drop quantified DNA was used for amplifying V3-V4 region of 16S using TAKARA ExTaq polymerase with specific primers which also have a tag sequence that were complementary to Illumina sequence adapter and index primers from the Nextera XT index kit V2. This round of PCR generated single amplicon of ~500–550 bp. Amplified products were checked on the agarose gel before proceeding for indexing PCR. In the next round of PCR (indexing PCR), Illumina sequencing adapters and dual-indexing barcodes were added to 25 ng (by Nanodrop) of amplified product using limited cycle PCR to give a final product of ~600–650 bp. All libraries after second round of PCR were normalized, quantified, estimated, and validated for quality by running an aliquot on High Sensitivity Tape Station-Agilent prior to sequencing on Illumina MiSeq (Illumina, San Diego, USA).

(i) V3-V4 amplification primers
  Read1:
  5′-TCGTCGGCAGCGTCAGATGTGTATAAGAGACAGCCTACGGGNGGCWGCAG-3′ Read2:
  5′-GTCTCGTGGGCTCGGAGATGTGTATAAGAGACAGGACTACHVGGGTATCTAATCC-3′
(ii) V3-V4 (Using Nextera XT Barcode Kit)

   Index2 Read: 5′-AATGATACGGCGACCACCGAGATCTACAC[i5]TCGTCGGCAGCGTC-3′

   Index1 Read: 5′-CAAGCAGAAGACGGCATACGAGAT[i7]GTCTCGTGGGCTCGG-3′

   wherein, W represents a degenerate base.

### Sequencing, de-multiplexing, and data pre-processing

The Illumina Miseq paired-end reads (250*2) were sequenced targeting an amplicon size of ~420–475 bases. The sequenced reads were de-multiplexed using Illumina bcl2fastq v2.17 tool allowing for 1 barcode mismatch in index1, and 2 barcode mismatch in index2 (in-house script). Raw (paired-end) sequence data (in fastq format) was initially pre-processed to perform end-clipping of reads with N’s at the end, and subsequently those reads were discarded that still contained (interspersed) N’s > 2%. Reads of low quality (minimum mean Phred score less than 25) and insufficient length (less than 100 bp) were also discarded. Subsequently, in each dataset, only those sequence pairs were retained in which both reads in a pair had individual mean quality greater than or equal to 25. Using the ‘join paired ends.py’ script available in QIIME^35^ (version 1.9.12), R2 reads were reverse complemented and merged with R1 reads (using default parameters). The resulting fasta files were then provided as input to V-Xtractor 2.0 program^36^ for retaining the V3-V4 region in each sequence. A summary indicating read-number statistics in groups of samples collected at various time-points of the study is provided as Supplementary Table S2.

### Taxonomic Assignments

Using the script (pick_otus.py) from QIIME version 1.9.1^35^, merged paired-end sequences were assigned using an open-reference OTU picking approach. Greengenes (version 13.8 having OTUs clustered at 97% identity) was employed as reference OTU database and UCLUST^37^ as the preferred OTU picking method (‘uclust_ref’ run with default parameters for clustering sequences with 97% identity). Reads obtaining hits to the reference OTU database (reference-OTUs) were assigned corresponding taxonomic lineages. Sequences that remained unassigned were clustered (at 97% identity) using *de novo* approach. This resulted in generation of 1686 *de novo* OTUs. Taxonomic assignments for resulting *de novo* clustered OTUs (*de novo*-OTUs) were obtained using MOTHUR ver. 1.29.2^38^. For this, sequences in each of the *de novo*-OTUs were provided as input to the Wang Classifier^39^ and assignments were done at 80% boot-strap threshold. Following the procedure as described in Ganju *et al*.^40^, the most common taxon was tagged to the respective *de novo* OTU. Taxonomic assignments could be obtained for 1451 of the original 1686 *de novo* OTUs. An abundance matrix comprising a total of 25,555 OTUs (including 24104 OTUs bearing correspondence to OTUs constituting the Greengenes database and 1451 *de novo* OTUs that could be assigned to a taxonomic lineage) was generated. Individual abundance matrices were also created based on the dosage amount (P, D1, D2, and D3) and study phase (Basal, Dosage, and Follow-up). Sparse OTUs i.e. those having zero abundance in >90% of samples (in each of the individual abundance matrices generated) were removed. Finally, a consolidated matrix comprising OTU abundance data for 621 samples corresponding to 69 participants (out of the initial 80), whose samples and corresponding sequence and associated metadata were available at all nine time-points of the study, were considered for downstream analysis (Supplementary File S2). OTU level abundances were also appropriately cumulated at higher levels of taxonomic hierarchy (phylum, class, order, etc.) as required for subsequent analyses steps.

### Statistical Analysis and Graph plotting

Unless otherwise mentioned, all statistical tests were performed using R (version 3.3.0). Box plots, bar-charts, line-plots, etc., were generated using ggplot ver. 2.0^41^.

## Results

A step-by-step analysis was carried out (on the generated taxonomic profiles) with the objective of obtaining answers to the following four questions -

1. What is the overall taxonomic structure of gut microbiota in the recruited participant cohort?
2. Do major gut microbial players change with dosage type/amount (D1, D2, D3, and P), and as per study phase (basal, dosage, and follow-up)?
3. Is there a significant change in gut microbiome community structure (in terms of species richness, evenness, and diversity)?
4. Does intake of FOS (or Maltodextrin) result in (a statistically significant) change in -
  - The abundance of known beneficial gut microbes
  - Level of random blood sugar, calcium, and triglycerides

Are changes (if any) sustained in the follow-up phase (i.e. post-discontinuation of dosage intake)?

### Gut Microbiota Profile of Recruited Participant Cohort

Analysis of taxonomic profiles across all time-points indicates a dominance of microbes belonging to four phyla, viz. *Bacteroidetes*, *Firmicutes*, *Proteobacteria*, and *Actinobacteria*. Supplementary Fig. S1 depicts the relative proportions of these phyla in the analysed datasets. The pattern of phylum-level assignments in the dosage and follow-up phases (across all time-points and dosage categories) appears to be more or less consistent with the basal-phase taxonomic profiles reported earlier^34^ for the same set of subjects. A comparison of the top 10 OTUs (in terms of median abundances across various study phases) indicates the prominent abundance of OTUs belonging to *Prevotella copri*, *Prevotella stercorea*, *Faecalibacterium prausnitzii*, and *Lactobacillus ruminis* in data sets across majority of study phases and dosage categories (Supplementary Table S3). In addition, OTUs belonging to *Sutterella*, *Oscillospira*, *Roseburia*, *Haemophilus*, *Dialister*, *Megasphaera*, *Turicibacter*, and *Bifidobacterium* are also observed to be present (although not that consistently). Most of the listed species are known to have beneficial effects on host gut metabolism^42–45^. Interestingly, OTUs belonging to *Bifidobacterium* appear in the Top 10 OTU list only during the dosage phase, and that too only in the specific subset of subjects who were administered Maltodextrin. This observation is in line with an earlier work^46^, which indicated the prolific growth of a majority of culturable strains of *Bifidobacterium* in Maltodextrin rich media (as compared to those in fructo-oligosaccharides).

### Community composition using Ordination Analysis

Taxonomic profiles across all time-points and dosage categories were visually analysed using Principal Coordinate Analysis i.e. PCoA (using Jensen-Shannon Divergence as the distance measure). Results of this analysis (depicted in Supplementary Fig. S2) indicate optimal grouping of microbiome sample profiles into two distinct clusters. The table (provided as inset in this figure) depicts the proportion of samples (belonging to each study phase and dosage type) in each cluster. If samples from basal phase are considered as belonging to ‘earlier’ time points, and those from dosage and follow-up phases together are considered as that belonging to ‘later’ time-points, then the pattern of distribution depicted in Supplementary Fig. S2 indicates the following. Majority (~80%) of earlier time-point samples are concentrated in Cluster 1. Although a higher proportion of samples (~62%) from later time-points are observed to still group together with earlier time-point samples in cluster 1, a sizeable subset of samples belonging to later time-points appear to segregate out into cluster 2. In spite of not having quite an overwhelming proportion, the size of the latter subset (~38%), as compared to the proportion of early-phase samples within the same cluster (~20%), indicates (albeit not so convincingly) that prebiotic intake does bring about a change in gut microbial community composition in a note-worthy proportion of participants. On a different note, the relatively higher concentration of most samples belonging to the basal and follow-up phases within a single cluster appears to indicate a reversal of the impact of prebiotics post-discontinuation.

### Pattern of alpha and beta diversity in the studied gut microbial communities

**Figure 2:**
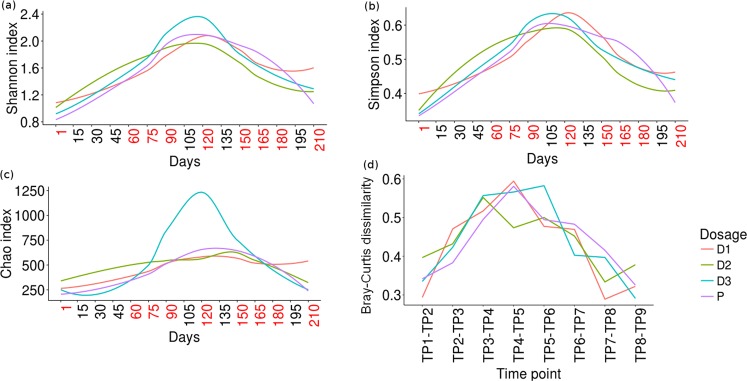
Alpha and beta diversity trends observed in microbiomes samples across various time-points and dosage categories. Changes in the gut microbial community structure in terms of the following 3 alpha-diversity measures (**a**) Shannon index, (**b**) Simpson index, and (**c**) Chao index. Prebiotic administration appears to cause a significant increase in alpha-diversity. Given that the interval between various sampling time-points was not equally spaced, the x-axis in panel’s (a–c) of the figure includes additional (non-sampling) time-points (indicated by black-coloured font). The LOWESS function in ggplot ver. 2.0^41^ was employed for plotting the trend-lines based on available values of alpha-diversity. A similar pattern is observed through employment of (**d**) Bray-Curtis dissimilarity - a beta-diversity measure that quantifies dissimilarity between microbial communities across successive time-points.

Three popular indices, viz., Shannon (for quantifying diversity), Simpson (a diversity measure that takes into account both richness and evenness), and Chao (for assessing community richness) were employed for analysing the effect of prebiotic intake (and subsequent withdrawal) on the overall structure of gut microbial communities in the studied participant cohort. Results of this analysis depicted as trend-lines (joining the pattern observed across various time-points, for each dosage type) indicate an unambiguous pattern (Fig. 2), wherein prebiotic intake/withdrawal (of any of the dosage type/amount i.e. D1, D2, D3, and P) appears to cause a corresponding increase/decrease (respectively) in overall community diversity, evenness, and/or richness. Intake of FOS at higher dosage (D3) particularly appears to cause a marked increase in community richness (Chao) as compared to other dosage amounts. The LOWESS function in ggplot ver. 2.0^41^ was employed for plotting the trend-lines based on available values that indicated the pattern for the respective diversity indices.

**Figure 3:**
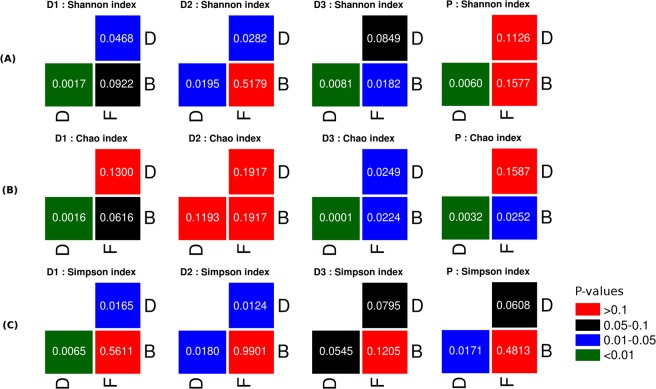
Statistical comparison of diversity metrics computed from microbiomes samples grouped by dosage type and study phase. Plot showing results of Mann-Whitney rank-sum test carried out between values obtained for an alpha-diversity measure/metric (e.g. Shannon). For each dosage type (D1, D2, D3, or P), the statistical test was carried out between. (i) set of metric values computed from all samples in the basal phase (B) vs. all samples in the dosage phase (D), (ii) set of metric values computed from all samples in the dosage phase (D) vs. all samples in the follow-up phase (F), and (iii) set of metric values computed from all samples in the basal phase (B) vs. all samples in the follow-up phase (F). For each of the four dosage types (D1, D2, D3, and P), three sets of p-values corresponding to the three types of comparisons viz. (i), (ii), and (iii) mentioned above were obtained for each of the three measures of alpha-diversity (depicted as horizontal panels A–C). Each horizontal panel therefore depicts four subfigures corresponding to results obtained with the four dosage type). In each sub-figure, the p-value obtained for the three comparisons viz. (i), (ii), and (iii) can be visualized and interpreted. For e.g. the top-right square (coloured blue) in the first sub-figure of panel (A) depicts the p-value obtained by statistical comparison between the set of metric values computed from all samples in the dosage phase (D) vs. all samples in the follow-up phase (F). The colours indicated in the legend provided for the figure can be used for interpreting the statistical significance of the p-value that was obtained.

Three approaches were further employed for evaluating the above observations (with respect to alpha diversity) from a statistical viewpoint. In the first approach, pair-wise statistical comparisons (employing Wilcoxon signed-rank test at fours levels of significance with Benjamini-Hochberg (BH) p-value correction) were performed between set(s) of values obtained for a given diversity metric at two (successive/non-successive) time-points. The four levels of significance considered were at (a) p-values < 0.01, (b) p-values 0.01–0.05, (c) p-values > 0.05 but less than equal to 0.1, and (d) p-values > 0.1. Results (Supplementary Fig. S3) depict that most pair-wise comparisons fail to achieve requisite statistical significance indicating that the observed distortion in the overall community structure (due to prebiotic intake/withdrawal) is quite likely a gradual community transition, rather than an abrupt change. In the second approach, for each of the dosage types/amount (D1, D2, D3, and P), Mann-Whitney rank-sum tests were carried out between (i) the set of metric values computed from all samples in the basal phase versus those obtained from all samples in the dosage phase (ii) set of metric values computed from all samples in the dosage phase versus those obtained from all samples in the follow-up phase, and (iii) set of metric values computed from all samples in the basal phase versus those obtained from all samples in the follow-up phase. Overall, results of these comparisons are depicted in Fig. 3. In most of the cases, intake of prebiotics appears to result in a statistically significant change with respect to diversity, richness, and evenness of gut bacterial communities. The third approach adopted was similar to the second, except that the statistical comparison was restricted to the set of alpha-diversity values that were computed from samples belonging only to the last two time-points in each phase. The idea was to check the long term effects on intake/withdrawal of prebiotic dosage. Results depicted in Supplementary Fig. S4 indicate a more or less similar pattern (as seen for the second approach). Furthermore, computing the medians of values obtained for each of the alpha-diversity metrics (in each of the compared dosage groups) indicates that samples from the dosage phase have higher median values (of alpha-diversity) as compared to that seen in samples from the basal phase.

Bray-Curtis dissimilarity scores were computed between samples (from individual subjects) at successive time-points to evaluate changes in beta diversity. Scores obtained between pairs of consecutive samples were plotted. Trend-lines joining the medians of such scores obtained between all such pairs of samples at successive time-points were generated for all dosage amount categories (Fig. 2). Results depicted add credence to the observation that intake of prebiotics results in a perceptible change in gut microbial community structure i.e. the taxonomic profiles get more dissimilar with prebiotic intake, and discontinuation of prebiotic intake causes the community to revert back to more or less its original composition thereby making the profiles relatively less dissimilar.

### Effect of prebiotic intake on *Bifidobacterium* and *Lactobacillus*

**Figure 4:**
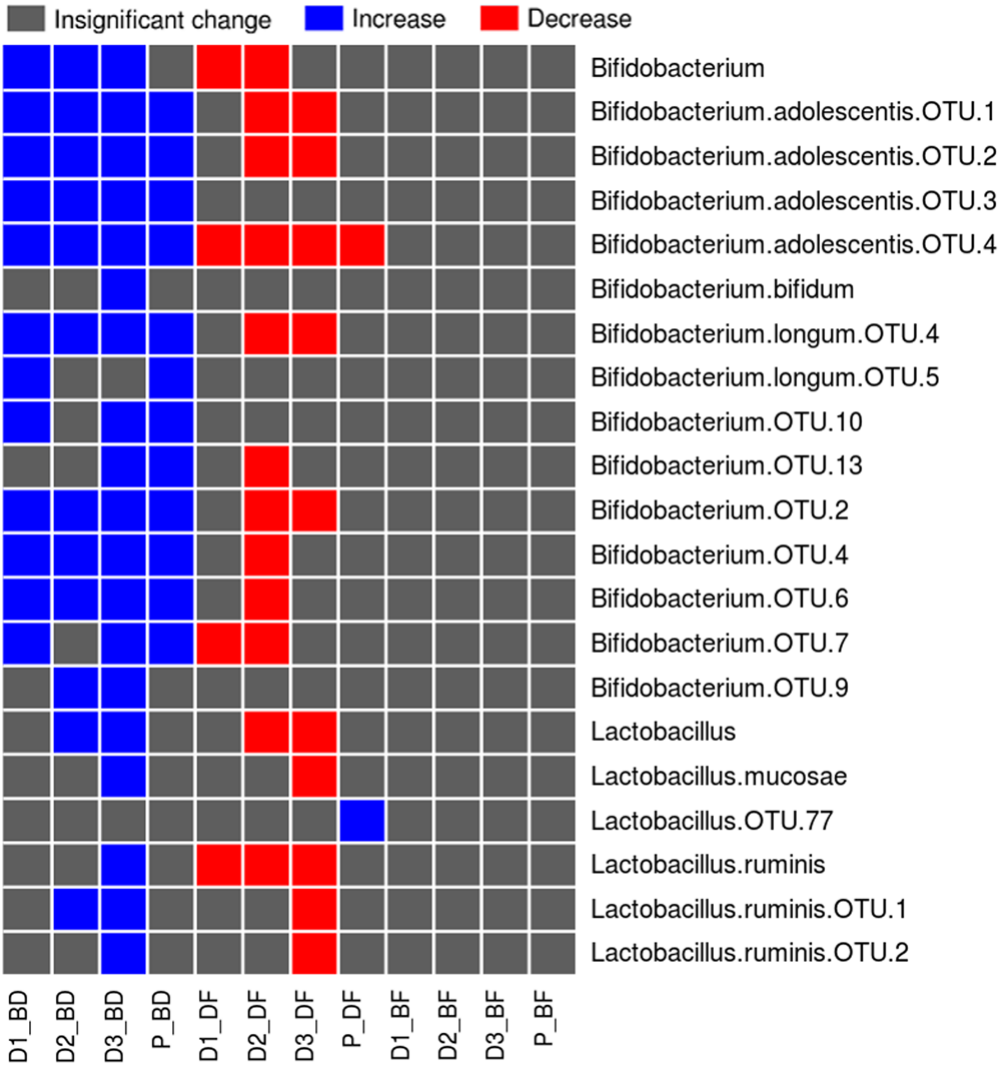
Abundance pattern of OTUs classified individually under *Bifidobacterium* and *Lactobacillus*. Results of Mann-Whitney rank-sum test carried out between percentage abundance values of *Bifidobacterium* and *Lactobacillus*. For each dosage type (D1, D2, D3, or P), the test was carried out individually for each OTU between (i) set of abundance values of a specific OTU computed from all samples of the basal phase (B) vs. all samples of the dosage phase (D), (ii) set of abundance values of a specific OTU computed from all samples of the dosage phase (D) vs. all samples of the follow-up phase (F), and (iii) set of abundance values of a specific OTU computed from all samples of the basal phase (B) vs. all samples of the follow-up phase (F).

Although *Bifidobacterium* and *Lactobacillus* are typically not amongst the most abundant genera found within human intestinal microflora, their probiotic and immuno-modulatory role during states of infection and inflammation has been highlighted by several studies^45,47–49^. Statistical analysis performed on the abundances of various OTUs (belonging to *Bifidobacterium* and *Lactobacillus*) across various time-points (TPs), study phases (basal, dosage, and follow-up), and with respect to dosage type/amount (D1, D2, D3, and P) indicates the following. Irrespective of type/amount, intake of prebiotics appears to result in a statistically significant increase in the abundance of most of the OTUs belonging to *Bifidobacterium* (Fig. 4). Interestingly, FOS (especially at higher dosage amount D3) appears to promote, in stark contrast to Maltodextrin, the selective (and statistically significant; Mann-Whitney rank-sum test) proliferation of OTUs belonging to *Lactobacillus* (Fig. 4).

A strange observation is also noticed with respect to the abundance pattern of a specific OTU i.e. OTU 77 belonging to *Lactobacillus*. Its abundance does not display any significant change (for all dosage types) between the basal to dosage (BD) and dosage to follow-up (DF) phases. However, its relative abundance indicates a statistically significant increase upon discontinuation of Maltodextrin (P_DF). This pattern seemingly indicates that Maltodextrin probably works in an antagonistic manner specifically with respect to the growth of this OTU of *Lactobacilllus*. Another similar interesting observation (with respect to abundance dynamics post-discontinuation of Maltodextrin) is with respect to various OTUs of *Bifidobacterium*. With the exception of OTU 4 of *Bifidobacterium*, all other OTUs belonging to *Bifidobacterium* do not display a significant decrease in their abundance after discontinuation of Maltodextrin intake. This however does not appear to be the case with FOS. Once FOS intake is discontinued, the abundances of most OTUs belonging to *Bifidobacterium* significantly decrease. Supplementary Fig. S5 depicts the abundance pattern of OTUs belonging to the genera Bifidobacterium and Lactobacillus.

### Dose-response relationship between amount of FOS intake versus abundances of *Bifidobacterium* and *Lactobacillus*

The amount of FOS intake (D1, D2, and D3) versus the median and spread of abundances of OTUs that were cumulatively classified under *Bifidobacterium* and *Lactobacillus* respectively (in samples belonging to all four time-points in the dosage phase) are depicted as box-plots in sections A and B of Supplementary Fig. S6. Results indicate a pattern similar to that observed in Fig. 4 wherein, in case of *Bifidobacterium*, dosage amount D3 (10gms of FOS) displays a relatively greater impact as compared to the other two dosage amounts (section A of Supplementary Fig. S6). Similarly, dosage amounts D2 and D3 (5 and 10gms of FOS respectively) appear to positively impact the growth of *Lactobacillus* during the dosage phase (section B of Supplementary Fig. S6). The mentioned changes/impact in the growth of Bifidobacterium and Lactobacillus (represented by cumulated abundances of OTUs belonging to the respective genera) are however not statistically significant (Mann-Whitney rank sum test).

In order to analyse differences between the extent to which FOS intake has an immediate impact as compared to the impact due to sustained FOS intake, box-plots were generated that represented, for various dosage amounts, the abundances of *Bifidobacterium* and *Lactobacillus* in TP2 (time-point in basal-phase which is just prior to commencing the intake of FOS), TP3 (i.e. after 15 days of FOS intake), and TP6 (after 90 days of FOS intake). Results of this analysis (depicted in sections C and D of Supplementary Fig. S6) indicate that while commencing FOS intake appears to result in an abrupt increase in the abundances of both *Bifidobacterium* and *Lactobacillus*, continued intake does not quite appear to sustain the initial impact. Even in this case, the increase in the cumulated abundances of Bifidobacterium and Lactobacillus (at the indicated time points) is not statistically significant (Wilcoxon paired rank sum test).

### Abundance pattern of specific taxa of interest (across various study phases)

Another objective of the study was to evaluate the effects of prebiotic intake on not only *Bifidobacterium* and *Lactobacillus*, but also other gut microbial species that are known to impact various aspects of gut metabolism. Two approaches were employed for this purpose. In the first approach, the cumulative abundance patterns of OTUs (individually assigned under the bacterial genera *Ruminococcus*, *Oscillospira*, *Sutterella*, *Paraprevotella*, *Bacteroides*, *Parabacteroides*, *Haemophilus*, *Succinivibrio*, and *Klebsiella*) were studied to check out if they demonstrated a statistically significant change in abundance with respect to dosage type/amount (D1, D2, D3, and P) and as per study phase (basal, dosage, and follow-up).

Results of this analysis are provided in Supplementary Fig. S7. Except for *Haemophilus*, most of these genera appear to be promoted by prebiotic intake (both FOS and Maltodextrin). In the second approach, the abundance pattern of these bacteria across all time-points was also plotted. Results of this analysis are depicted in Supplementary Fig. S8. In this figure, the LOWESS function in ggplot ver. 2.0^41^ was employed for plotting the trend-lines based on values that indicated the pattern of median abundances of the depicted genera (in the four participant groups) at various time-points. Results depicted in Supplementary Fig. S8 indicate interesting patterns. While *Faecalibacterium* and *Ruminococcus* display abundance patterns (across time-points) similar to that seen for *Bifidobacterium*, the abundances of genera *Sutterella* and *Oscillospira* also show an increasing trend after discontinuing the intake of prebiotics. The abundance patterns obtained with other genera are inconsistent and eliciting any meaningful inferences from the observed patterns is difficult. For instance, the abundance of *Roseburia* appears to plummet for a brief period during the dosage phase, post which its abundance appears to increase/stabilize. Although the overall trend of *Roseburia*’s abundance is not quite consistent (as compared to that observed for few other genera like *Faecalibacterium* and *Ruminococcus*), the brief (yet sharp) dip in abundance during the dosage phase (and the later increase/stabilization) is likely due to competition for resources created by other co-occurring genera (e.g. *Ruminococcus*) that perform similar functions as *Roseburia*. This competitive trend, however, appears to be a transient phenomenon and (as seen) on a longer time-scale, *Roseburia* appears to retain/regain its rightful/optimal abundance that justifies its role as a commensal, thereby ensures its permanence in the human gut^50,51^.

Although the pattern of abundances depicted in Supplementary Fig. S8 visually indicate a heightened abundance of genera like *Sutturella* and *Oscillospira* in the follow-up phase (as compared to basal phase), the temporal abundance pattern (depicted in Supplementary Fig. S7) for various species/OTUs belonging to these genera does not indicate a statistically significant change. It may be noted that the two mentioned analyses were performed on data at two distinct levels of taxonomy (one performed at the level of OTUs, and the other on cumulative abundances of individual OTUs belonging to a taxon at genus level). While in most cases, results of both analysis indicated concordance, for a few genera (e.g. *Oscillospira*) the results were inconsistent. This could possibly be either a visual artefact due to biases arising due to the different means of analysis or probably an effect of the relatively low abundance values observed for these genera.

### Impact of prebiotic intake/withdrawal on levels of triglyceride, calcium, and serum glucose

**Figure 5:**
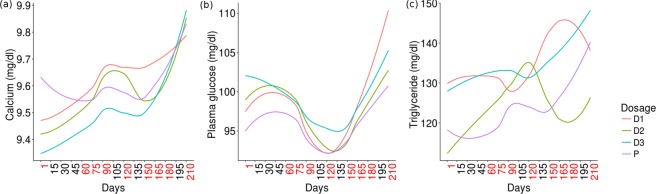
Trends observed with respect to biochemical test parameters monitored in the study participant cohort. Pattern of changes in biochemical test parameters:(**a**) calcium, (**b**) plasma glucoseand (**c**) triglyceride levels respectively. Given that the interval between various sampling time-points was not equally spaced, the x-axis in panels a-c of the figure includes additional (non-sampling) time-points (indicated by black-coloured font). The LOWESS function in ggplot ver. 2.0^41^ was employed for plotting the trend-lines based on available values of alpha-diversity.

**Figure 6:**
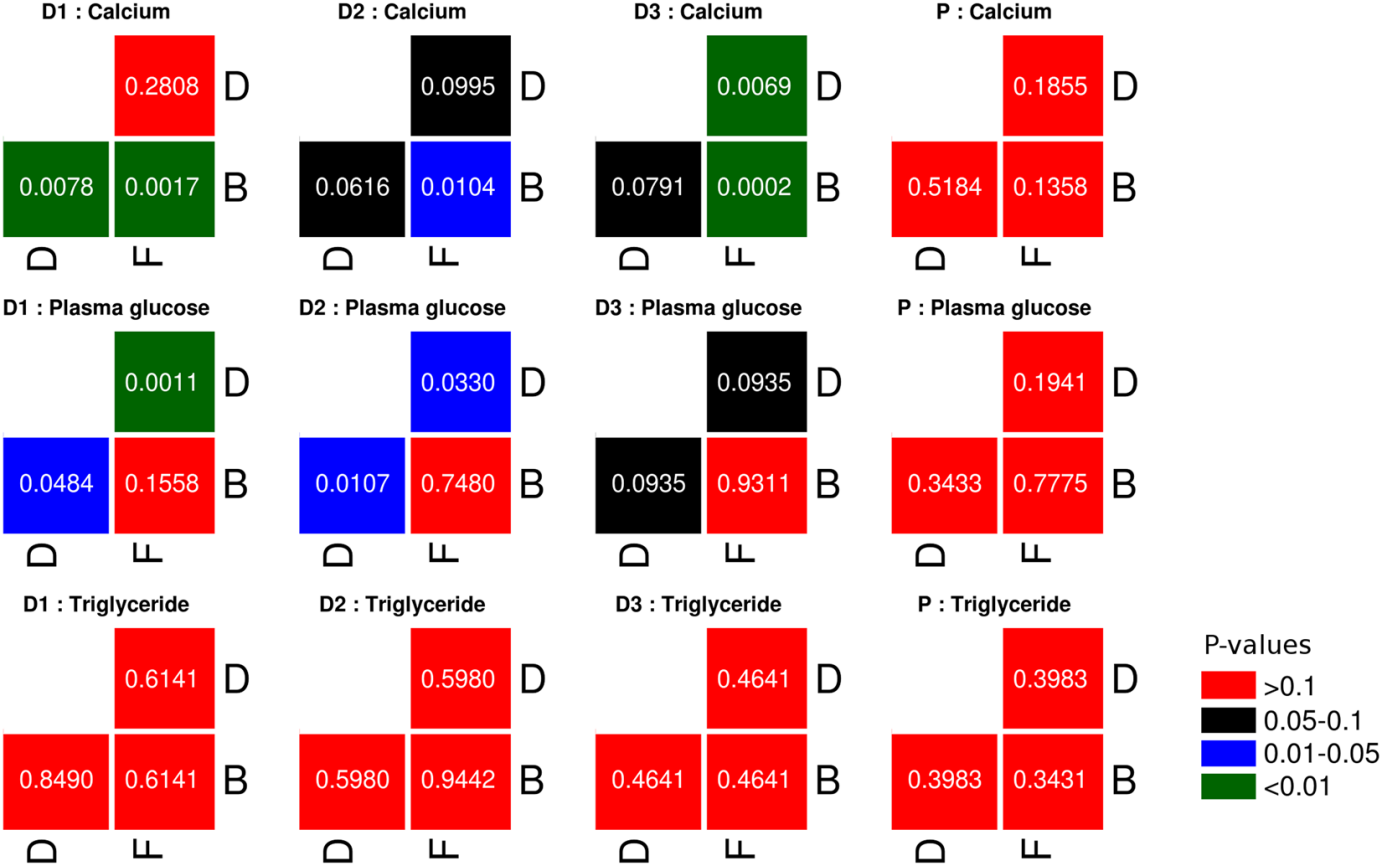
Statistical comparison of individual biochemical test parameters (grouped by dosage type and study phase). Results of Mann-Whitney rank-sum test carried out between values obtained for an individual biochemical test (e.g. triglyceride). For each dosage type (D1, D2, D3, or P) the test was carried out between (i) Set of values obtained from study participants belonging to a dosage type in the basal phase (B) vs. corresponding values in the dosage phase (D), (ii) Set of values obtained from study participants belonging to a dosage type in the dosage phase (D) vs. all samples in the follow-up phase (F), and (iii) Set of values obtained from study participants belonging to a dosage type in the basal phase (B) vs. all samples in the follow-up phase (F).

The three statistical analysis approaches adopted for studying changes in community structure due to prebiotic intake were also adopted for studying the effects of prebiotic intake and withdrawal on three biochemical parameters, viz. serum triglycerides, calcium, and glucose. Results of this analysis are depicted in Figs 5 and 6 and Supplementary Figs S9 and S10.

Primarily, it is observed that almost all values for all the three biochemical parameters lie within normal healthy reference ranges. Specifically, it is interesting to note that prebiotics (both FOS and Maltodextrin) appear to positively impact (albeit in miniscule proportions) the levels of serum calcium (Fig. 5). However, the abruptly increasing trend of serum calcium post-discontinuation of prebiotic intake is quite an intriguing phenomenon (Fig. 6). Results depicted in Supplementary Figs S9 and S10 however indicate that statistically significant changes with respect to elevated levels of serum calcium are only with FOS intake (at specific dosage amounts), and not with Maltodextrin. A more-or-less similar pattern (although as an inverted trend) is observed with respect to plasma glucose. The patterns of results obtained with respect to serum triglycerides are quite random and none of the statistical tests done with respect to their values across various dosage phases indicate any statistical significance for both FOS as well as Maltodextrin. It may be noted that analysis to check if the abundances of any of the OTUs shows a correlation with the abundances observed for triglycerides, calcium, and blood glucose did not yield any statistically significant correlations.

## Discussion

The last couple of decades have witnessed significant changes with respect to the global pattern of food consumption^52–54^. Besides a marked structural shift from basic staples towards more diversified diets, the food intake of a majority of the global urban populace (and to some extent their rural counterparts) currently includes foods with relatively higher energy density, convenience/junk foods, sugar-sweetened beverages, sports drinks, etc. This shift in dietary pattern coupled with increased sedentary life-styles is contributing to a higher prevalence of health issues, primarily metabolic disorders^55^. Several recent studies have implicated dysbiosed gut microbiota as one of the prime drivers behind various mechanisms that contribute to the development and manifestation of metabolic disorders^56–59^. On a different note, there is emerging clinical evidence indicating (a) an inverse relationship between incidence of metabolic disorders and the appropriate intake of a fibre-rich diet, and (b) the favourable role that prebiotic dietary fibres play in maintaining/restoring the gut microbiota in/to a balanced (healthy) state^7–9,26–31^.

FOS (Fructo-oligosaccharides) has been one of the most researched dietary fibre (prebiotic) supplements. There have been a few recent studies (involving human participants) that have clinically evaluated the impact of FOS on the global composition of gut microbiota^26–29^. Although, results obtained in these studies primarily (re)confirm the Bifidogenic properties of FOS, aspects such as (a) the effects of variable quantities of FOS supplement on gut microbiota, and (b) the temporal dynamics of gut microbiota (transitioning through basal, dosage, and follow-up phases) have never been studied in detail. The experiments performed in the present study represent an attempt to understand the above mentioned aspects.

Increased microbiome biodiversity, in most cases, has been shown to correspond with a relatively “healthier” gut state. Increased diversity is likely to heighten the probability of existence of functionally related microbes which can efficiently (a) collaborate by sharing functions/metabolites required for expressing a given functionality, or (b) compensate for the presence/absence of missing species in case of stresses caused due to internal/external influences.

Results obtained in this study (Figs 2 and 3) indicate that prebiotic intake does result in a significant increase in bio-diversity of the gut microbiome. A rather simplistic analysis of lists corresponding to non-sparse taxa present in basal and/or dosage phases (across all dosage types/amounts) indicates an approximate addition of 15% newer taxa in the dosage phases as compared to that present in the basal phases. Interestingly, almost the entire set of non-sparse taxa found in the basal phase is retained in the dosage phase. This observation in a way indicates that intake of prebiotics (i.e. those used in the present study) does not disrupt/alter the original composition of the gut microbiome, but only augments its existing taxonomic repertoire. Using tools that facilitate inferring functions from 16S data, it will be interesting to analyze whether the changes with respect to the taxonomic repertoire (identified in samples across various time-points/phases of the study) also bring about a change (and in what manner) in the functional potential of microbial communities^60–64^. However, on a different note, these results also necessitate re-confirmation through further experimentation and analysis given that increase in diversity is an opposite trend to the findings of a recent study which indicated/hypothesized that increased uptake of dietary fibre (via. prebiotics or specialized diets) might lead to a biased enrichment of specific fibre-digesting microbes thereby leading to a temporary state of reduced microbial diversity^65^.

Within the dosage phase, the observed pattern of increase in community diversity and richness (depicted in Fig. 2) is rather intriguing. Diversity and richness appear to peak around Days 105–120, after which it starts declining. Although a definitive reason for this observation cannot be proposed, resilience of complex microbial communities to major and sustained perturbations could possibly be a cause behind this phenomenon^66–68^. Investigating such resilience and recovery patterns in the context of prebiotics administration will however require a study design that incorporates a relatively longer time period of not only the dosage phase but also in the follow-up phase. In this context, it may be noted that although a few earlier studies have probed the long-term effect of prebiotics^28,69–72^, the scope of these studies has been restricted to monitoring the growth dynamics of only specific microbes of interest (i.e. *Bifidobacterium* and *Lactobacillus*). The pattern of diversity, specifically at a community level (and moreover at different dosage quantities), has not been investigated in as much detail as attempted in the present study.

On a different note, hydrogen, carbon dioxide and SCFAs (primarily acetate, butyrate, propionate) are typically known end products of carbohydrate catabolism by colonic bacteria^73^. End products of this fermentation process additionally include lactic acid which is produced by species belonging to Bifidobacterium as well as Lactobacillus. The mentioned end products, in addition to their beneficial role in inhibiting/restricting the growth of entero-pathogenic microbes and/or microbes that promote harmful putrefactive processes, also lead to acidification of colonic environment. It may be hypothesized that, over a period of time, the resulting acidification contributes to autologous repression of further growth and proliferation of the beneficial microbes themselves (including Bifidobacterium and Lactobacillus). The latter phenomenon might be the likely reason for pattern of abundances depicted in panels C-D of Supplementary Fig. S6. The figure depicts the lack of a sustained (enhancing) impact of FOS intake (during the dosage phase) with respect to the abundances of Bifidobacterium and Lactobacillus in the analysed microbiome samples.

Results, with respect to the significant increase in the abundance of most OTUs belonging to the genus Bifidobacterium after intake FOS as well as Maltodextrin (Fig. 4), appear to be consistent with the pattern of results obtained in a few earlier studies^74,75^. These intervention (culture-based) experiments were however carried out with infants as study participants. Although the results of the mentioned studies lack statistical significance, analysis of faecal samples collected from these infants (fed with milk supplemented with prebiotics) demonstrates a noticeable increase in the levels of both Bifidobacteria and Lactobacilli along with concomitant reduction in the growth of a few pathogenic species (e.g. Clostridium)^74,75^. In a different study^76^, experiments carried out over a period of 12 weeks, with 48 healthy adults as participants, oligo-fructose supplementation resulted in reduced body weight (1.03 ± 0.43 kg). Interestingly, the control group in these experiments were observed to gain additional body weight (0.45 ± 0.31 kg; p = 0.01). With respect to plasma glucose levels, while participants in the intervention group demonstrated a decrease in the measured levels, the control group showed a statistically significant (p ≤ 0.05) increased level in the final test when compared to that taken at the beginning of the experiment. Results of a recent study^77^, wherein the faecal microbiome was studied (in lactose intolerant individuals) pre-administration and 30 days after stopping of GOS intake, also indicated a statistically significant increase in the abundance of *Bifidobacterium*, *Faecalibacterium*, and *Lactobacillus* in 90% of the participants (27 out of 30).

Another interesting titbit from our analysis is the specific presence of 47 OTUs (with a minimum cumulative assigned read count of 10,000) in greater than 80% of the entire set of 621 samples analyzed in the present study (in all three study phases, irrespective of dosage intake/discontinuation, in whatever dosage amounts or types). Amongst these 47 OTUs, the following 19 obtained classification at the level of genus or below. *Prevotella copri*, *Faecalibacterium prausnitzii*, *Lactobacillus ruminis* OTU_1, *Roseburia faecis*, *Haemophilus parainfluenzae* OTU_47, *Prevotella stercorea*, *Streptococcus* OTU_207, *Prevotella*, *Ruminococcus gnavus*, *Sutterella* OTU_8, *Lactobacillus ruminis*, *Dialister* OTU_12, *Turicibacter* OTU_4, *Mitsuokella multacida*, *Bifidobacterium*, *Roseburia*, *Megasphaera* OTU_4, *Bifidobacterium adolescentis*, *Klebsiella* OTU_17, and *Mitsuokella*. Most of these microbes are known for their beneficial butyrogenic properties as well as other known functions^78–83^. Results of statistical analysis also indicate that apart from probiotic bacteria (*Bifidobacterium* and *Lactobacillus*), a significant change was also observed in certain butyrate-producing microbes like *Faecalibacterium*, *Ruminococcus* and *Oscillospira*. *Faecalibacterium* and *Ruminococcus* are known butyrate-producers, but *Oscillospira* has been gaining this status recently. The latter is known to degrade host glycans, i.e., they are dependent on the metabolites produced by other fibre-degrading microbes and hence produce SCFA from otherwise non-digestible glycans in human intestine^43^.

In this context, it is important to note here results appear to demonstrate the ability of FOS to selectively stimulate the growth of specific intestinal bacteria that are known to positively impact the health and well-being of an individual. This specific ability is in-fact represents one of the three criteria that needs to be fulfilled prior to labelling any food ingredient as a prebiotic^10,84^. Results obtained in this study appear to indirectly imply that FOS also satisfies the other two criteria, namely, the ability to withstand the acidic environment of the stomach and escape degradation/hydrolysis by digestive enzymes, and subsequent availability of the prebiotic as a substrate amenable for fermentation by microflora in the small intestine. It may however be conceded that the present study design does not incorporate specific experiments, the results of which would act as direct evidence regarding the two criteria mentioned above.

FOS is not broken down by human digestive enzymes. Moreover, there are no scientific studies establishing the differential impact of FOS consumed before or after the meal. However, since food generally neutralizes the pH of the stomach, an advantage of supplementing FOS along with or after food would reduce the hydrolysis due to the acidic environment. We therefore hypothesized that a post-dinner prebiotic intake would contribute to its increased availability for fermentation by colonic microflora. On a different note, consumption of the prebiotic prior to a meal may given rise to undesirable adverse effects like bloating, the latter being a likely result of rapid fermentation of the prebiotic fibre in the small intestine. However, given the limited understanding of these processes at this point of time (especially pertaining to the optimal timing of taking a prebiotic), the above explanations are merely hypotheses and cannot account for observed individual variations (if any). Moreover, the stoichiometry of the fermentation process is also expected to be a function of the chain-length and the monosaccharide composition of the administered prebiotic^73^.

In addition to demonstrating the (statistically significant) Bifido/Lactogenic effects of FOS and Maltodextrin, Fig. 4 also indicates interesting trends with respect to the “reversal of accrued changes” upon discontinuation of prebiotic intake. The trends with respect to the transition between Dosage and Follow-up phases (indicated as ‘DF’ in the figure) indicate a statistically significant decrease in the overall abundance levels (for a subset of OTUs) of *Bifidobacterium* as well as *Lactobacillus*. Viewed in the context of results (of comparison between the abundance levels of OTUs corresponding to these two bacterial species) between Basal and the Follow-up phases, and other results obtained with respect to community diversity, a definitive restoration of the original microbiome state is quite apparent once the participants discontinue the intake of the evaluated prebiotics. Whether these observations open up possibilities/opportunities for targeted (favourable) microbiome manipulation in clinical settings remains to be seen.

Several recent studies have reported the beneficial impact of *Bifidobacterium* and *Lactobacillus* in terms of increasing/lowering the levels of serum calcium and glucose, respectively^85–87^. The significantly increased levels of OTUs belonging to these two microbes (Fig. 4) in the dosage phases thereby add credence to the pattern observed in these studies and appears to explain the observed rise/dip in serum calcium and glucose levels respectively during the dosage phase (Fig. 5). However, given that the sample collection protocol (particularly with respect to blood samples) did not take into specific consideration the effects of possible confounding factors such as variability in diet taken by individual participants (specifically on the day of sample collection), the variations observed particularly with respect to values of random blood sugar need to be evaluated/considered keeping this perspective in mind. The abruptly increasing trend of all three studied biochemical parameters post-discontinuation of prebiotic intake is however quite an intriguing phenomenon (Fig. 6). It will be interesting to observe the follow-up phase trends over a longer time-scale than that performed in the present study.

Different classes of prebiotic compounds are expected to demonstrate variability with respect to their mode of action, extent/capability of modulating the levels of serum lipids. It is also logical to expect that the type/quantum of SCFAs produced by different colonic microbes determine the extent of their impact on the level of circulating lipids. For instance, the conversion of acetate to acetyl-CoA and the availability of the latter as a substrate for fatty acid synthesis in hepatocytes explain the increase in levels seen for cholesterol and other serum triglycerides in experiments involving rectal infusions of acetate^88,89^. Given this, GOS and Lactulose (substrates that primarily produce acetate) are likely to contribute to increase in serum lipid levels, upon consumption^89^. On the other hand, FOS is reported to produce more or less similar proportions of both butyrate and acetate, which therefore sort of counterbalances the lipogenic effects of acetate^89^. Fermentation of fructo-oligosaccharides also produces propionate which, in turn, inhibits/prevents lipid synthesis that is induced by acetate^88,89^. Overall, the said processes could be one amongst the several likely reasons for the statistically insignificant changes that are observed with respect to the level of triglycerides upon FOS intake (Fig. 6).

While the study period corresponding to the dosage phase was long enough (with 4 time-points spanning 3 months) to observe any significant changes, this phase ideally should have been of a higher duration to rightly ascertain the long-term effects of Maltodextrin as compared to FOS. The reason for this is the following. Maltodextrin is known to possess a glycemic index between 85–105 as compared to FOS, the corresponding index of which falls in the range between 0 and 1^86^. Earlier studies have indicated that a long term intake of Maltodextrin is associated with high serum glucose levels^90^. It would have been interesting to assess if Fructo-oligosachharides also display a similar impact on long-term usage. Furthermore, in theory, this study lacks a true placebo arm which would have captured time-dependent influences (if any) to the abundances of relevant OTUs in this population. This aspect is an important consideration to be kept in mind in future studies.

## Supplementary information

Supplementary Information

Supplementary File S2: OTU abundance dataset

## Data Availability

The 16S rRNA sequences generated in this study have been deposited into the European Nucleotide Archive with accession number PRJEB28572. The corresponding metadata information is provided as Supplementary File S3.
