# Supplementary material for: A prospective randomized, double-blind, placebo-controlled, dose-response relationship study to investigate efficacy of fructo-oligosaccharides (FOS) on human gut microflora
Source: Sci Rep. 2019 Apr 2;9:5473. doi: 10.1038/s41598-019-41837-3 (PMC6445088; doi:10.1038/s41598-019-41837-3)
Supplement: Supplementary file 1 — Supplementary Information [file 41598_2019_41837_MOESM1_ESM.pdf]

Supplementary information

for

A prospective randomized, double-blind, placebo-controlled, dose-response relationship study to investigate efficacy of fructo-oligosaccharides (FOS) on human gut microflora

Disha Tandon[1], Mohammed Monzoorul Haque[1], Manoj Gote[2], Manish Jain[2], Anirban Bhaduri[2], Ashok Kumar Dubey[2], Sharmila S. Mande[1*]*

[1] *Bio-Sciences R&D Division, TCS Research, Tata Consultancy Services Ltd., 54-B, Hadapsar Industrial Estate, Pune 411 013, Maharashtra, India (DT, MMH, SSM)*

[2] *Tata Chemicals Ltd. Innovation Centre, Survey Number 315, Hissa Number 1-14, Ambedveth, Mulshi, Pune 412111, Maharashtra, India (MG, MJ, AB, AKD)*

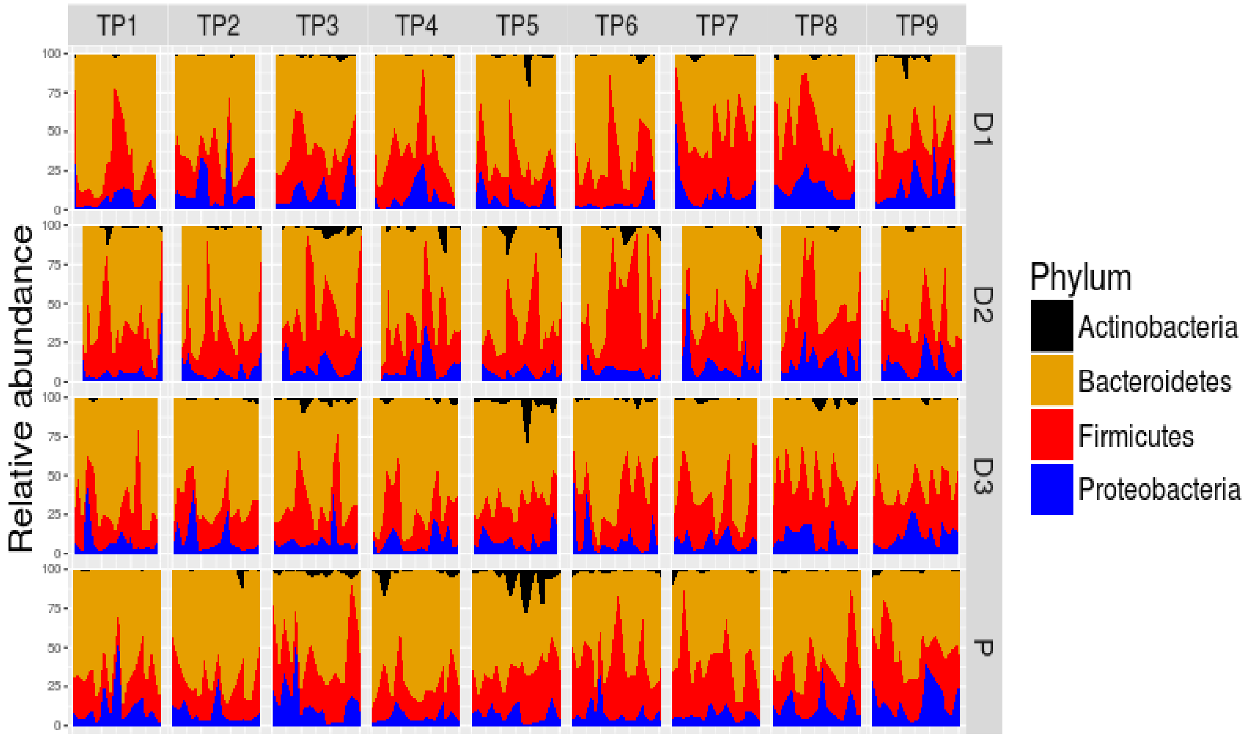

Supplementary Figure S1: Phylum-level taxonomic distribution pattern
Area curve representing the relative abundance of four major phyla in all samples across various time points and phases of the study. The figure was generated using microbiome taxonomic profiles corresponding to 69 study participants. Microbiome sequence data could be generated from stool samples provided at all 9 time-points of the study for these 69 participants.

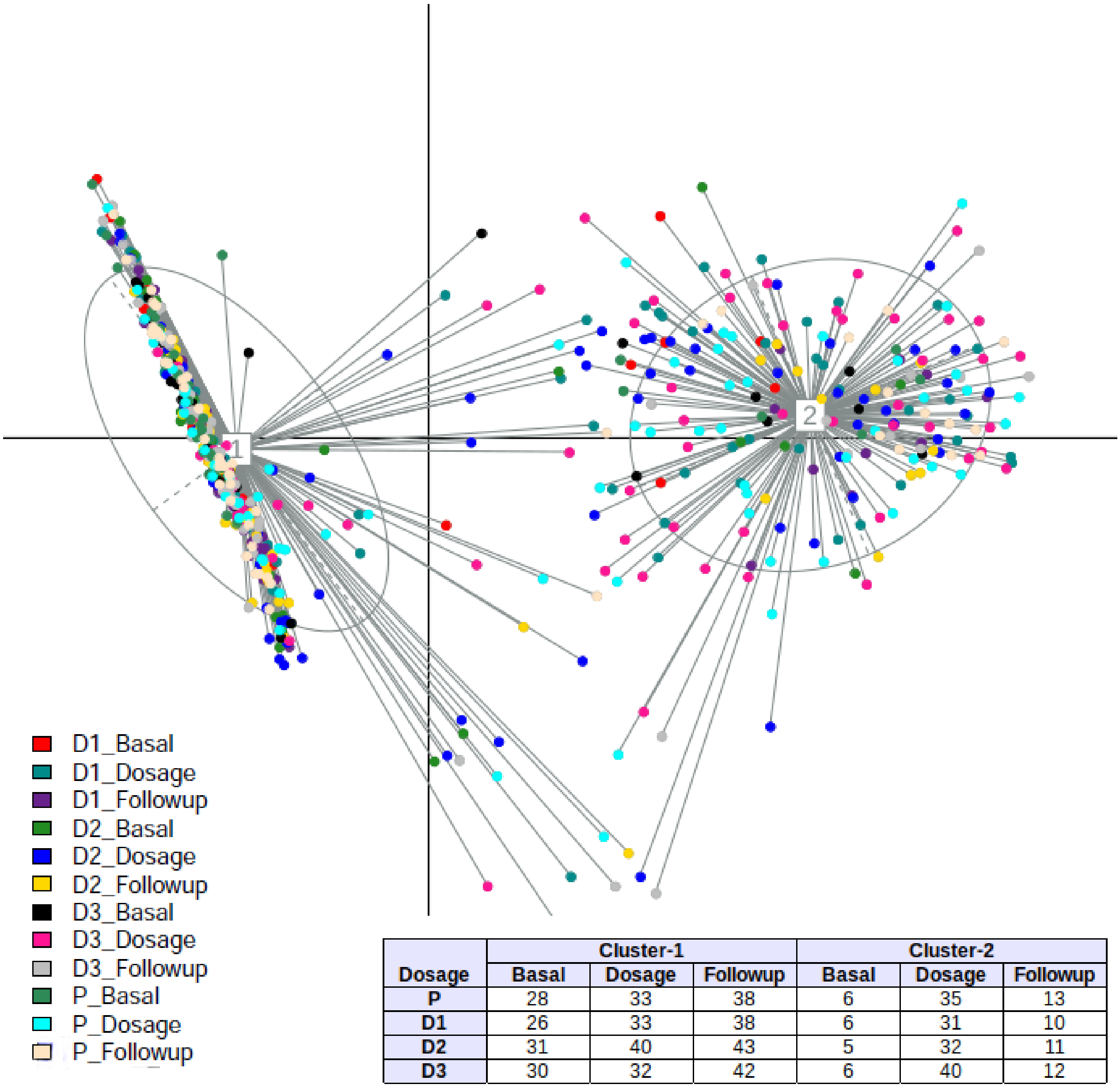

Dosage	Cluster-1			Cluster-2		
	Basal	Dosage	Followup	Basal	Dosage	Followup
**P**	28	33	38	6	35	13
**D1**	26	33	38	6	31	10
**D2**	31	40	43	5	32	11
**D3**	30	32	42	6	40	12

Supplementary Figure S2: PCoA clustering of microbial abundance data based on Jensen Shannon divergence

Two distinct clusters were obtained. The inset depicts the numerical distribution of samples in the two clusters. Samples from basal and follow-up phases appear to be relatively more concentrated in the first cluster indicating that discontinuation of prebiotic intake restores the gut microbial community to its original state.

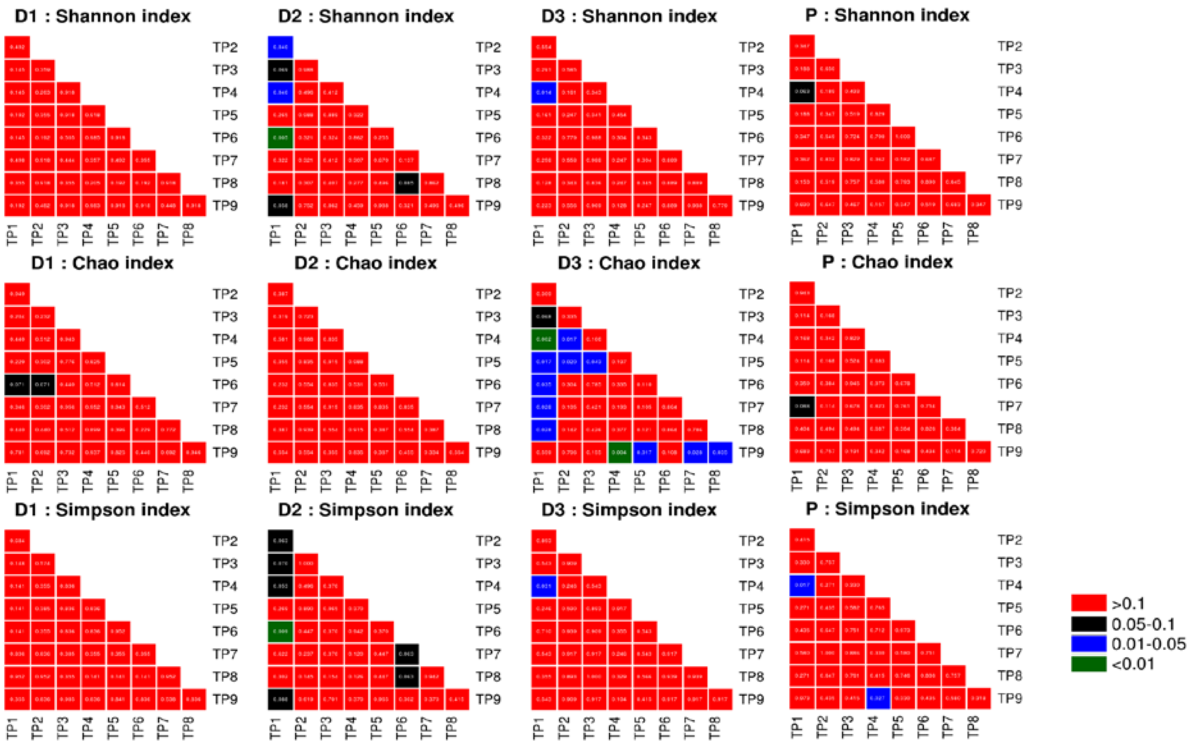

Supplementary Figure S3: Statistical comparison of diversity metrics computed from microbiomes samples (from 69 subjects) across successive/ non-successive time-points and dosage categories

Plot showing results of Wilcoxon paired rank-sum test carried out between values obtained for an alpha-diversity measure/ metric (e.g. Shannon). The test was carried out between a set of metric values obtained from samples belonging to a particular time point, and the corresponding (paired) set of values obtained from samples (taken from the same study participants) at another time-point.

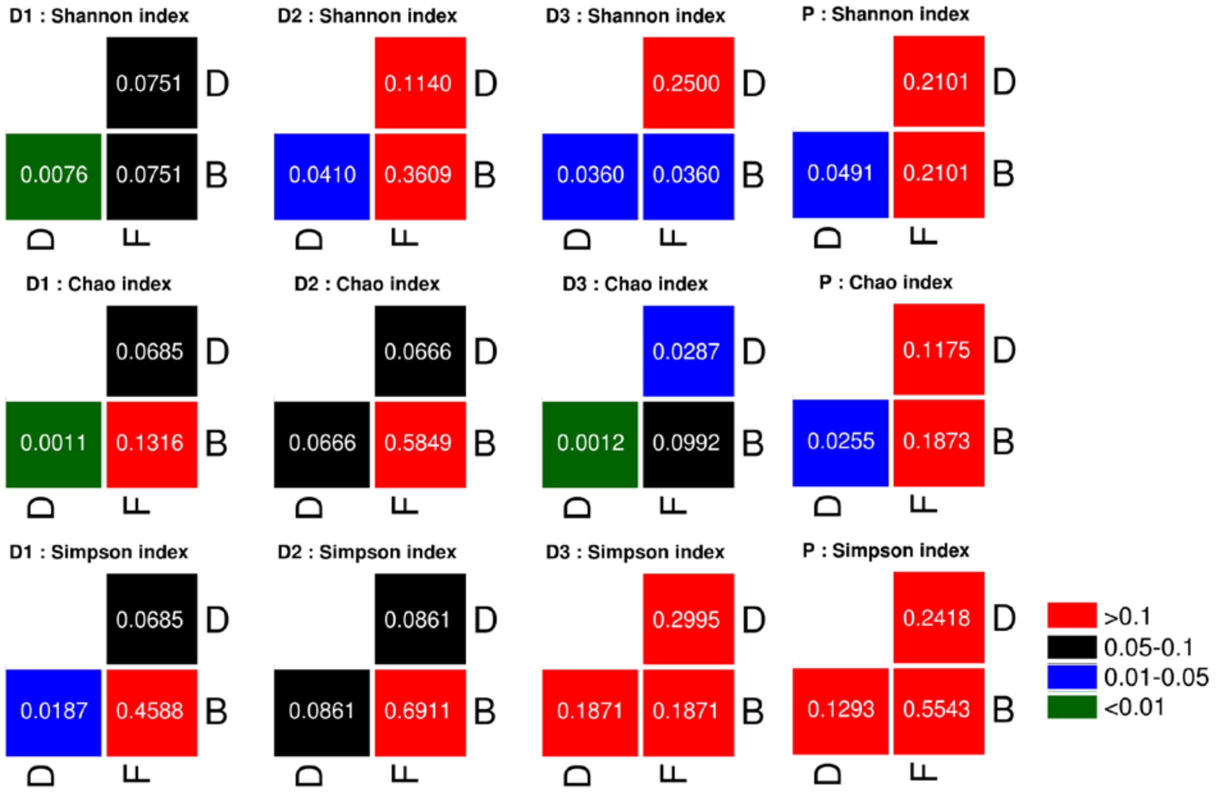

Supplementary Figure S4: Statistical comparison of diversity metrics computed from microbiomes samples grouped by last two time-points in each study phase

Plot showing results of Mann-Whitney rank-sum test carried out between values obtained for an alpha-diversity measure/ metric (e.g. Shannon). For each dosage type (D1, D2, D3, or P) the test was carried out between -

(i) set of metric values computed from samples taken in two time-points of the basal phase (B) vs. samples taken in last two time-points of the dosage phase (D),

(ii) set of metric values computed from samples taken in last two time-points of the dosage phase (D) vs. samples taken in last two time-points of the follow-up phase (F), and

(iii) set of metric values computed from samples taken in two time-points of the basal phase (B) vs. samples taken in last two time-points of the follow-up phase (F).

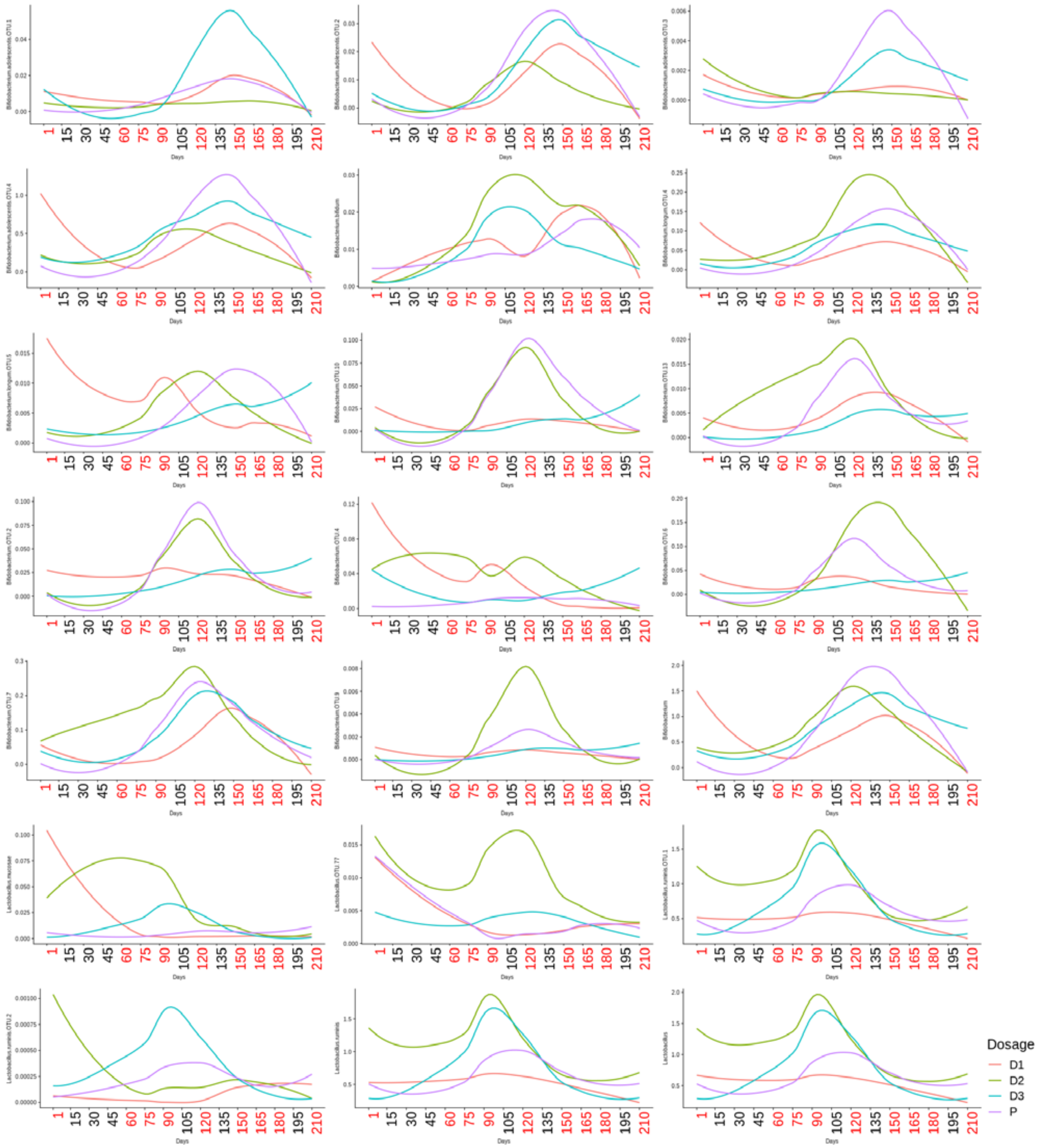

Supplementary Figure S5: Abundance pattern of OTUs belonging to Bifidobacterium and Lactobacillus.

Plots depicting the pattern of abundance values of OTUs belonging to the genera Bifidobacterium and Lactobacillus. Image incorporates the patterns for all four dosage categories viz., D1, D2, D3, and P. Given that the interval between various sampling time-points was not equally spaced, the x-axis in each sub-figure include additional (non-sampling) time-points (indicated by black-coloured font). The LOWESS function in ggplot ver. 2.0 was employed for plotting the trend-lines based on available abundance values.

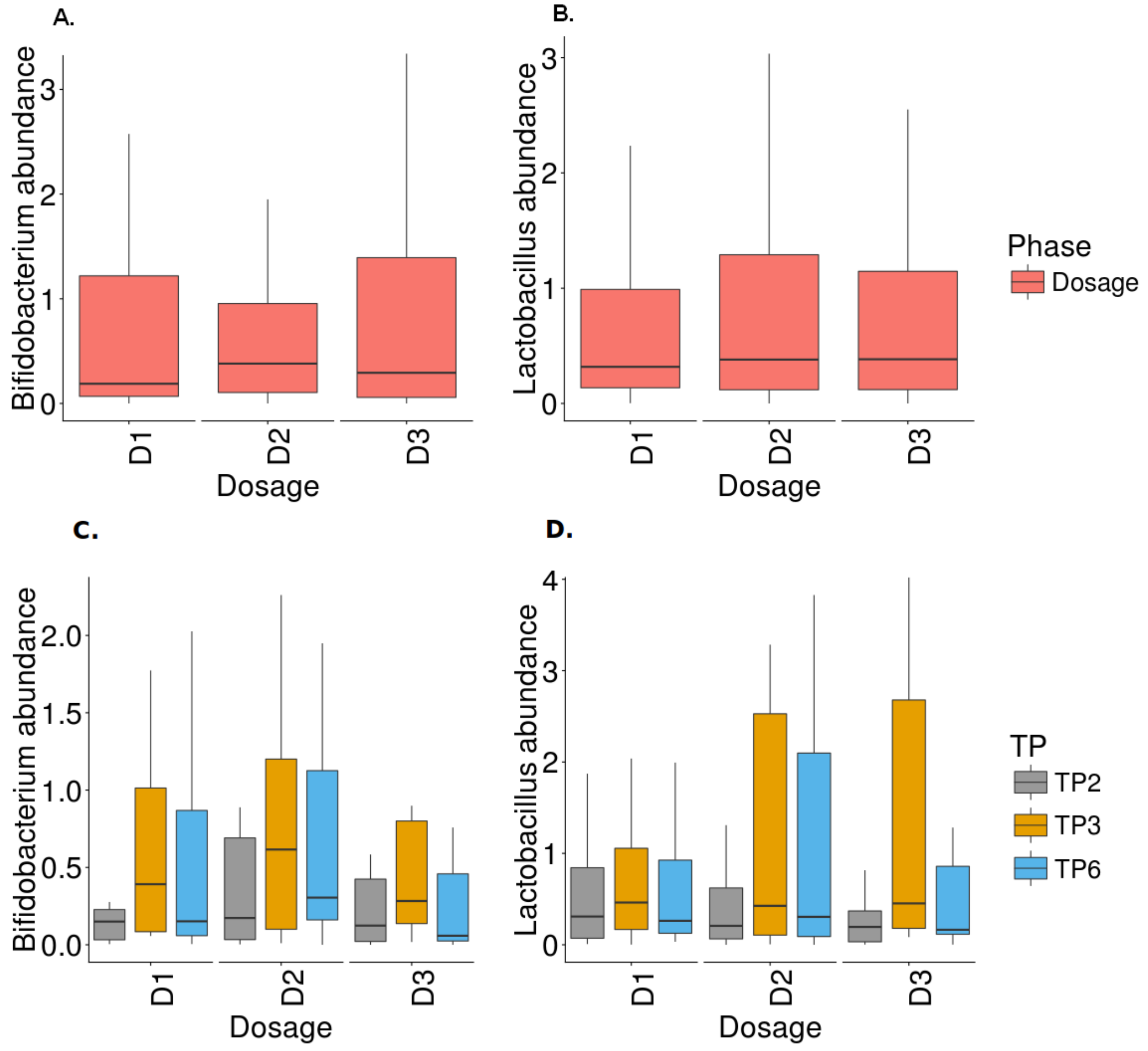

Supplementary Figure S6: Dose-response relationship for Bifidobacterium and Lactobacillus. Panels A and B depict box plots that indicate the median and spread of abundances of OTU's belonging to Bifidobacterium and Lactobacillus and their relationship with dosage amount (D1, D2, and D3). Also depicted in the lower half of the image (panels C and D) are box-plots that indicate the noticeable increase of these two beneficial bacteria as the study moves through to the dosage phase (end of TP3), and also after a sustained period of prebiotic administration (end of TP6).

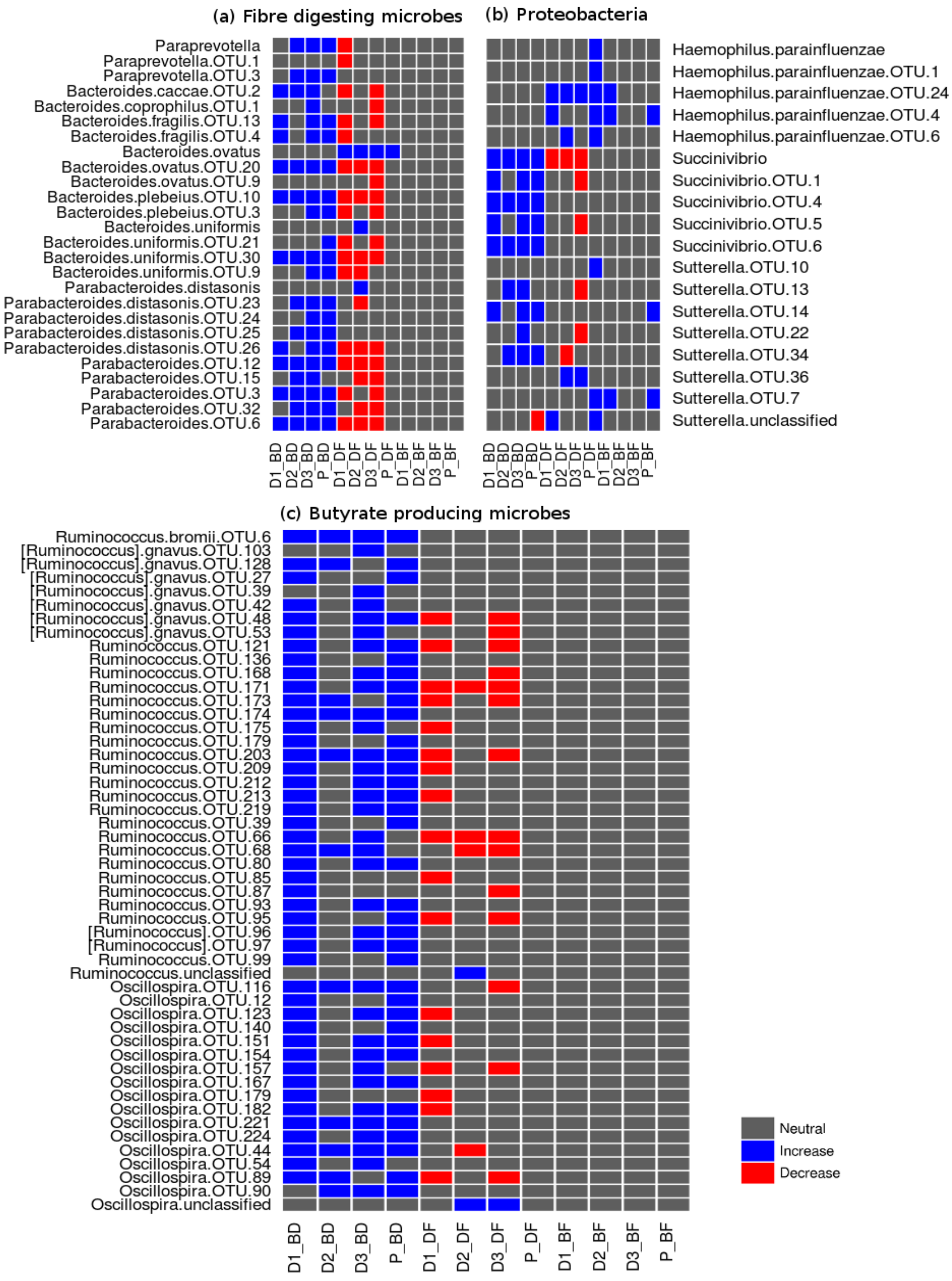

Supplementary Figure S7: Results of statistical tests evaluating the abundance pattern of OTU's classified to other gut bacterial taxa known to impact gut metabolism

Plot depicts results of Mann-Whitney rank-sum test carried out between percentage abundance values of other gut bacterial taxa known to impact gut metabolism. For each dosage type (D1, D2, D3, or P), the test was carried out individually for each OTU between

(i) Set of abundance values of a specific OTU computed from all samples of the basal phase (B) vs. all samples of the dosage phase (D),

(ii) Set of abundance values of a specific OTU computed from all samples of the dosage phase (D) vs. all samples of the follow-up phase (F), and

(iii) Set of abundance values of a specific OTU computed from all samples of the basal phase (B) vs. all samples of the follow-up phase (F).

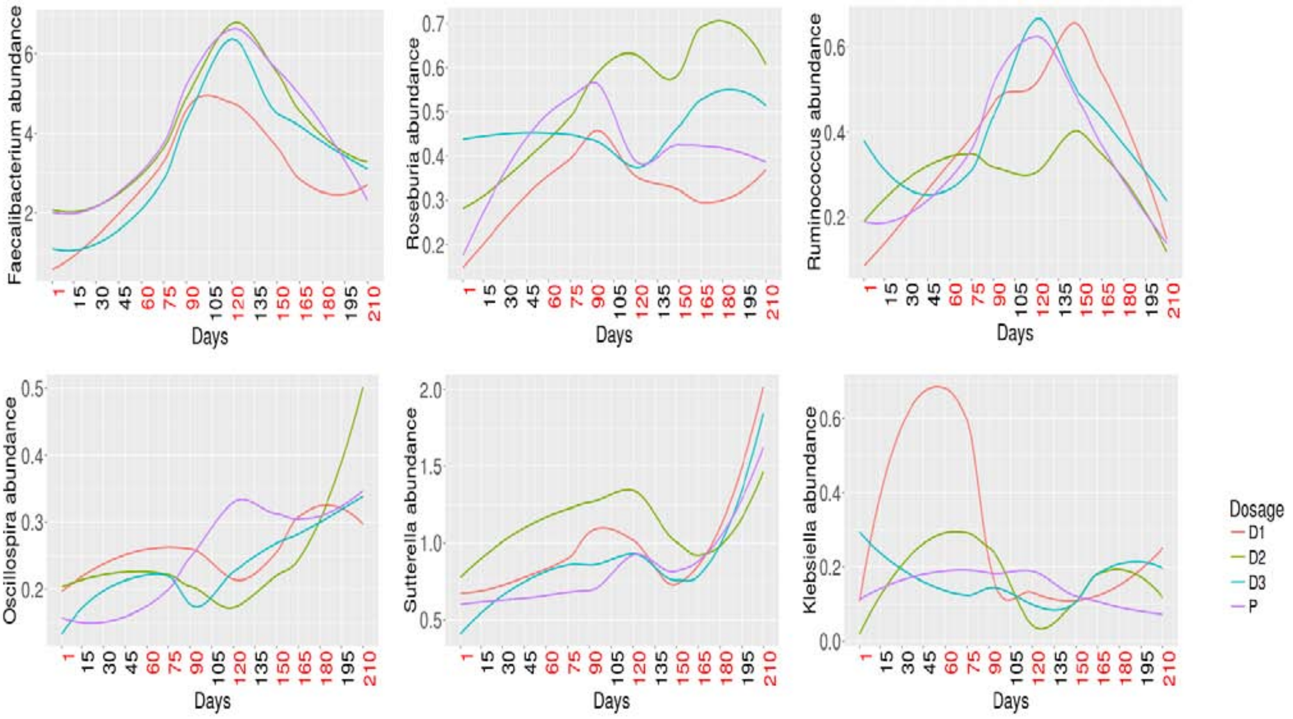

Supplementary Figure S8: Abundance pattern of other gut bacterial taxa known to impact gut metabolism

Plots depicting the pattern of abundance values of other gut bacterial taxa known to impact gut metabolism. Image incorporates the patterns for all four dosage categories viz., D1, D2, D3, and P. Given that the interval between various sampling time-points was not equally spaced, the x-axis in all sub-panels of the figure includes additional (non-sampling) time-points (indicated by black-coloured font). The LOWESS function in ggplot ver. 2.0 was employed for plotting the trend-lines based on available abundance values.

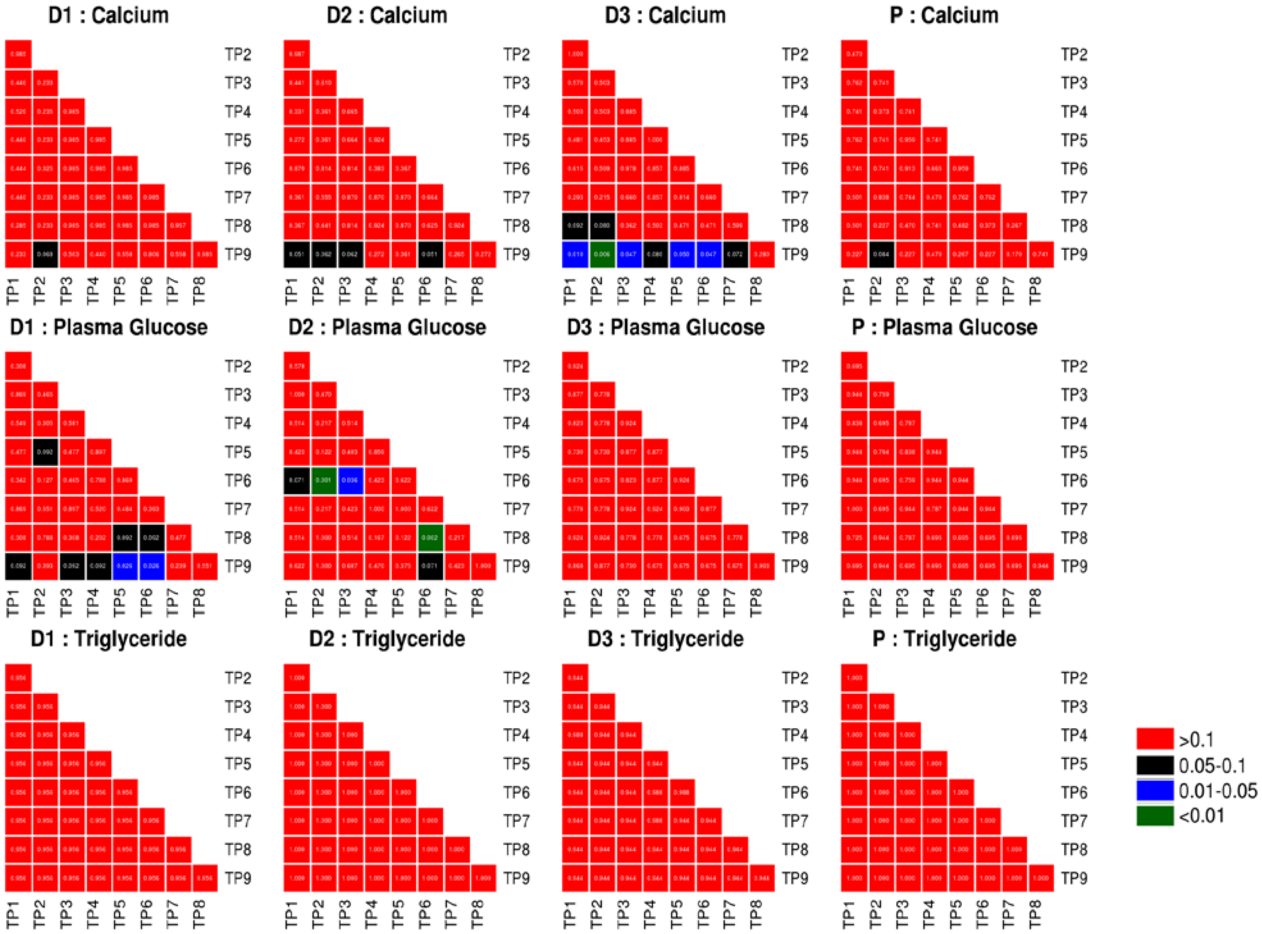

Supplementary Figure S9: Statistical comparison of individual biochemical test parameters across successive/ non-successive time-points and dosage categories

Plot showing results of Wilcoxon paired rank-sum test carried out between values obtained for a biochemical test (e.g. triglyceride). The test was carried out between a set of values from study participants at a particular time point, and the corresponding (paired) set of values (taken from the same study participants) at another succeeding time-point.

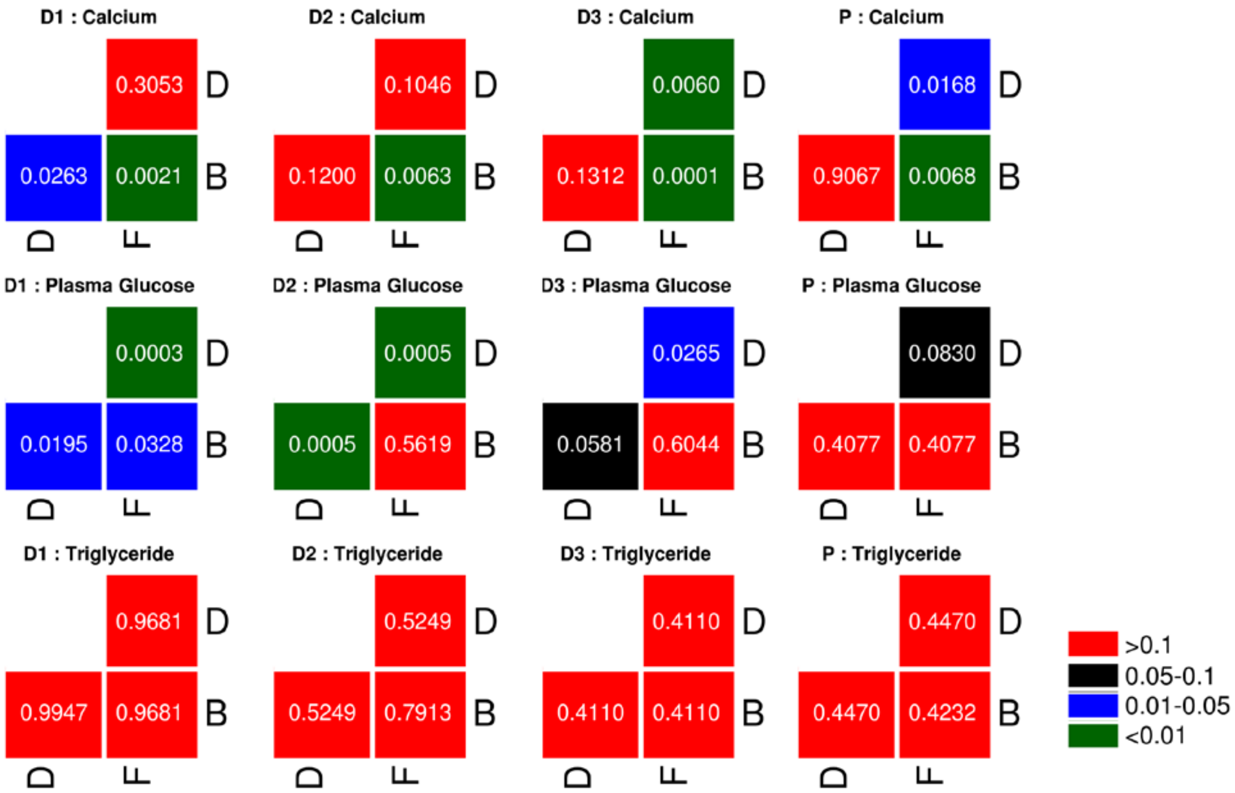

Supplementary Figure S10: Statistical comparison of individual biochemical test parameters grouped by last two time-points in each study phase and dosage type

Plot showing results of Mann-Whitney rank-sum test carried out between values obtained for a biochemical test (e.g. triglyceride). For each dosage type (D1, D2, D3, or P) the test was carried out between -

(i) Set of values obtained from study participants in two time-points of the basal phase (B) vs. values taken in last two time-points of the dosage phase (D),

(ii) Set of values obtained from study participants in the last two time-points of the dosage phase (D) vs. values taken in the last two time-points of the follow-up phase (F), and

(iii) Set of values obtained from study participants in two time-points of the basal phase (B) vs. values taken in last two time-points of the follow-up phase (F).

Supplementary Table S1: Metadata corresponding to participants enrolled in the present study

A spread-sheet providing details of metadata corresponding to study participants. The sheet also includes results of the three biochemical tests that were performed on the blood samples collected from the study participants at all 9 time-points of the study.

Subj. ID	Sex	Asian	Diet (V – Veg; NV - NonVeg)	Age (Yrs)	Height (cm)	Weight (Kg)	BMI (Kg/m^2)	Pre/ Post menopausal	Habits	Dosage	Plasma glucose									Triglyceride									Calcium								
											Day-1	Day-60	Day-75	Day-90	Day-120	Day-150	Day-165	Day-180	Day-210	Day-1	Day-60	Day-75	Day-90	Day-120	Day-150	Day-165	Day-180	Day-210	Day-1	Day-60	Day-75	Day-90	Day-120	Day-150	Day-165	Day-180	Day-210
1	Male	Y	V	30	176	78.6	25.37			D1	90.6	92.2	90.9	90.7	87.1	92.5	75.2	115.3	94.8	173	86.8	176.5	96.4	142.3	88.3	199.9	198.5	163.5	9.3	9.5	9.9	9.9	10	9.6	9.9	9.9	9.6
2	Male	Y	NV	33	168	74.5	26.4		Tea [Taken 3 before days]	P	100.7	112.3	91.4	103.4	100.8	90.5	90.3	92.4	113.6	134.1	71	80.2	60.6	111.5	112.6	81.1	57.4	135.4	9.6	9.5	9.3	9.8	9.3	9.5	9.6	9.6	10.1
3	Male	Y	NV	29	170	71	24.57		Pan Masala (Non Tobacco) [Left 4 months back]	D2	100.1	96.2	83.1	85.1	85.1	83.8	96.3	101.5	99.5	127.7	108.7	88.4	111.9	110.5	125.5	106	110.2	107.9	9.8	9.5	9.5	9.9	9.5	9.6	9.8	10	9.7
5	Male	Y	NV	32	175	74	24.16			D3	99.6	88.7	104	97.8	97.9	96.4	98.6	91.9	105.8	224.4	165.6	234.8	183.6	190.3	201.1	190.8	245.7	240.6	9.9	9.8	10.1	10.1	10.4	9.9	9.9	10	10.4
6	Male	Y	V	33	164	62.2	23.13			D2	120.9	101.1	120.9	133.7	137.5	93.5	134.8	97.4	142.2	211	240.3	235.5	240.9	243	119.9	394.2	309	244.3	9.7	10.3	9.9	10	10.1	9.9	10.1	10.1	10.2
8	Male	Y	NV	27	157	60	24.34		Tea (one cup/ day)	P	84.6	118.6	138.6	95.1	108.9	89.3	80.4	116.3	93.9	139.7	203.9	195	190.9	152.2	140.1	260.9	240.7	134.3	11.2	10.1	10.3	10.1	10.3	10.3	10.9	10.4	10.3
9	Male	Y	NV	32	153	50	21.36			D2	86.9	86.6	95.1	87.5	90.3	90.6	96.9	96	82.6	157.4	134.8	209.8	152.5	173	193.2	155.9	105.6	185.2	10.1	8.9	9.5	9.6	9.2	9.2	9.5	9.1	10
10	Male	Y	V	41	167	73	26.18		Non tobacco pan masala; (2/ day; Left 6 months ago)	P	85.7	95.7	95.7	87.9	82.4	91.6	128.3	148.8	92.7	193.5	115	141.2	234.7	172.3	240.1	157.9	288.5	205.1	9.6	9.3	9.2	9.4	9.4	9.6	9.3	9.9	9.7
11	Female	Y	NV	30	147	46.5	21.52	Pre	Tea (one cup/ day)	D3	93.1	86.2	86.9	77.2	79.1	92.2	83.6	87.4	97.7	58.9	78.4	57.5	41.2	53.8	71.8	68.4	68.7	67	10.2	9.9	9.6	10.3	9.9	10.1	9.9	10.3	10.1
12	Female	Y	NV	33	146.5	53	24.69	Pre	Tea (one cup/ day)	D1	92.9	106.9	103.4	95.9	94.6	89.1	96.7	117.2	125.5	97.8	175.3	147.4	159.5	124.8	133	174.3	121	92.3	9.1	9.1	9.3	9.5	9.4	9.4	9.3	9.3	9.5
14	Female	Y	NV	39	155	49.3	20.52	Pre		D1	100.5	119.8	113.9	109.2	105.2	102.6	116.5	114.1	131.9	107	64	88.2	77.2	56.9	65.3	96.1	76.8	68	9.5	9.2	9.6	9.8	9.5	9.8	9.3	9.2	9.4
17	Female	Y	V	42	162	74.5	28.39	Post	Tea (two cups/ day)	D3	116.7	106.1	107.9	107.6	102.5	111.3	104.9	103.7	132.6	239.1	239.1	241.4	237.3	225.1	239.3	331.1	224.2	238.9	9.3	9.2	9.3	9.9	9.8	9.7	9.6	9.8	9.6
19	Female	Y	V	33	154	70	29.52	Pre		P	93.7	111.3	110.7	114.8	82.7	90.4	100.8	126.6	124.1	75.6	104.1	93.6	75.9	84	64.8	135.2	149	104.9	9.2	9.1	9.3	8.9	9.3	9.2	9.5	9.3	9.7
20	Female	Y	V	28	157	65	26.37	Pre		D2	91.5	96.3	98.7	103.3	70.6	92.1	90.2	98	103.1	94.4	63.8	84.2	76.5	155.2	113.5	89.2	73.6	107.1	9.7	9.3	9.6	9.6	10.3	9.6	9.5	9.5	10.3
22	Male	Y	V	40	173	81	27.06		Non tobacco pan masala (2/day); Left since 3 years	D3	133.3	97.5	100.3	112	127.6	110.4	102.8	93.1	123.3	194.3	157.5	139.5	137.3	133.5	108.5	141.6	125.9	159.5	9.6	9.9	9.8	9.6	9.7	9.5	9.9	9.8	9.6
23	Male	Y	NV	30	163	62.8	23.64		Tea (one cup/ day)	P	100	128.6	110	80.7	95.9	103.3	107.5	102.8	107.2	80.7	103.1	195	58.1	224.5	100.1	84.3	106.2	198.1	9.4	9.4	9.9	9.6	9.2	9.6	9.1	9.7	9.9
24	Male	Y	NV	29	157	45.8	18.58		Tea (one cup/ day)	D2	97.6	102.8	98.2	100.5	82.6	91.4	76.7	102	103.7	63.3	61	74.3	74	134.6	62	62.1	72.6	66.7	10.1	9.8	10	9.9	9.7	10.1	10.2	10.2	10.2
25	Male	Y	NV	40	163	54.8	20.63			D3	86.8	111.4	100.4	111.8	91.6	77.8	87.9	109.7	99.1	136.4	222.4	214.5	224.5	167.7	162.1	198.5	192.9	216.7	9.4	9	9.5	9.6	9.3	9.2	9.5	9.6	10.1
26	Male	Y	NV	38	170	56.5	19.55			D2	82.3	86.5	91.3	83.1	74.8	80	69.3	90.5	73.6	80.3	82.2	57.5	124.4	74.7	58.5	119.5	78.4	90.2	9.3	9.6	9.4	9.5	9.8	9.1	9.5	9.3	9.8
27	Male	Y	NV	29	170	55.3	19.13		Tea (one cup/ day)	D1	90.8	100	94.8	84.8	89.2	93.1	97.9	88.8	95.5	44.5	48.1	50	49.7	52.1	59.8	61.8	46.2	64.2	9.8	9.6	9.4	9.5	9.7	9.6	9.5	9.7	10
29	Male	Y	NV	41	164	62.3	23.16			D2	108.8	128.2	112.2	112.5	103.3	110.1	113.2	115	122.2	193	166	143.2	205	171.7	178.7	170	201.3	175.9	8.9	9	9	9.7	8.9	9.1	8.9	9.6	9.5
30	Male	Y	NV	40	164	73.5	27.33		Tea (one cup/ day)	D3	90.8	85.3	85.7	82.1	80	118	89.3	88.7	104.9	142.5	113.9	115.4	134.3	173.4	196.9	229.1	149.4	165.6	9.7	9.6	9.7	9.7	9.8	9.8	10.1	9.8	10
31	Female	Y	V	28	155	70	29.14	Pre	Tea (one cup/ day)	D1	101.9	130.5	120.9	119.3	104.9	110.6	93.5	117.6	123	87.7	127.3	125.7	110.6	135.2	148.8	148.1	177.8	107.2	9.6	9	9.9	9.6	9.7	9.8	9.8	10.1	10
32	Female	Y	V	23	147	49	22.68	Pre	Tea (one cup/ day)	P	97	96.5	90.9	93.5	85.4	87.3	89	95.8	98.1	62.3	125.7	121.2	130.7	160.9	100.3	89.9	74.9	73.6	9.3	9.6	9.7	9.7	9.8	9.5	9.4	9.5	9.6
33	Female	Y	NV	37	152	47.7	20.65		Tea (one cup/ day)	D2	93.6	97.2	93.3	97.3	79.8	82.2	78.6	99.1	91.9	44.7	53.8	53.1	33.3	62.8	51.3	34.8	49.6	44.9	9.2	9.2	9.5	9.2	9.3	9.1	8.9	9	9.9
34	Female	Y	NV	37	150	63.8	28.36	Pre	Tea (one cup/ day)	D3	100	101.5	93.8	77.8	88.7	79.7	73	82	78.8	105	104.9	149.4	133.9	57.4	109.8	118.6	81.3	114	9.3	9	9.3	9.7	8.8	10	9.4	9.2	9.8
35	Female	Y	NV	37	151	52	22.81	Pre	Tea (one cup/ day)	P	112.4	101.5	105.1	97.5	101.5	106.9	98.6	110.6	117	63.1	88.6	101.6	91.2	75.6	75.4	83.1	99.3	91.2	9.5	9.1	9	9.4	9.1	9.2	9.3	9.3	9.4
36	Female	Y	NV	34	149	45.8	20.63	Pre	Tea (one cup/ day)	D1	89.4	90.4	95.6	84.1	80.6	81.4	88.4	87.5	76.8	62.6	129.7	96.9	92.9	93.5	96.5	69.7	109.9	57.1	9.6	9.4	9.6	9.5	9.6	9.8	9.5	9.2	9.6
37	Female	Y	V	40	150	67	29.78	Pre	Tea (two cups/ day)	D3	99.5	102.8	100.8	100.3	110	91.5	112.8	93.8	127.9	82.4	89.5	169.3	138.1	107.3	114.8	77.5	92.8	179	9.6	9.3	9.7	9.9	9.4	10	9.8	9.7	10.3
38	Female	Y	NV	35	149	55	24.77	Pre	Tea (one cup/ day)	P	80.1	87.6	85.9	82.3	94.3	86.2	82.9	77.8	83.4	95.8	129.6	149.6	232.3	151.4	147.3	129.8	176.7	215.5	9.4	9.1	9.3	10	9.4	9.4	9.1	9.2	9.7
39	Female	Y	NV	35	147.5	60.6	27.85	Pre	Tea (two cups/ day)	D2	112.8	106.7	104.3	104.7	110.8	91.5	136.3	105.7	118.7	81.6	90.9	122.7	121.2	169.8	158.4	86.4	115.6	134.8	8.9	9.8	9.8	9.9	9.5	9.8	9.6	9.8	10.2
40	Female	Y	NV	35	153	68	29.05	Pre	Tea (two cups/ day)	D1	94.1	93.9	90.4	88.2	92.2	87.6	88.8	92.5	107.6	157	172.6	102.3	181.5	157.6	145.7	152.1	145.1	134	10.1	10.2	9.8	10.1	9.8	10	10	9.9	10.1
41	Male	Y	NV	36	167.5	83.5	29.76			D1	107.1	107.1	98.9	109.4	92.8	110.4	104.7	101.6	110.7	219	254.4	148.4	175.6	142.5	243.8	263	154.5	214.7	8.9	9.6	9.7	9.6	9.3	9.5	9.9	9.6	9.7
42	Male	Y	NV	39	157	55.6	22.56		Tea (one cup/ day)	P	87.3	99	82.1	79.8	107	104.9	95.8	110.3	97.1	114.2	135.1	129.2	154.9	187.6	209.7	225.3	141.8	190.3	9.6	9.4	9.6	9.7	9.5	9.6	9.8	10.3	10.2
43	Male	Y	NV	34	161	56	21.6		Non tobacco pan masala (2/day; Left 12 months ago)	D3	91.1	100.9	91.8	97.2	79.7	92.6	86.1	98.1	102	83.2	89.3	67.7	81.5	103.2	76.9	47.7	113.3	106.3	9.2	9.3	9.4	9.2	9.3	9.1	9.4	9.1	9.7
44	Male	Y	V	30	188	67.5	19.1		Tea (two cups/ day)	D2	100.4	99.1	96.1	87.7	86.1	84.7	135.8	137.7	97.2	45.2	115.7	60.8	78.9	73.1	164.8	62.5	60.8	65.2	9.5	9.6	9.8	10.1	9.8	9.6	9.4	9.4	10.1
45	Male	Y	NV	39	168	63	22.32			D1	87.8	81.9	85.1	83.3	84.7	71.7	90.9	75.2	95.2	212.2	127.2	188.1	59.8	84.5	159.4	119.3	248.7	217.6	9.1	9.2	9.5	9.5	9.1	9.2	9.3	9.4	9.6
46	Male	Y	NV	35	161.5	68.2	26.15		Tea (one cup/ day); Non tobacco pan masala; (left since 4 years)	D3	136.3	99.6	98.6	96.1	108.5	102.1	91.7	122.1	91.6	73.9	60.1	69.8	83.1	111.3	142.1	116.9	142.3	221.1	9.4	9.3	10.3	9.7	9.6	9.6	9.7	9.8	9.9
47	Male	Y	NV	40	165	52	19.1		Non tobacco pan masala (1/day; Left 12 months ago)	D2	111.4	104	114.6	94.4	100.8	83.6	94.4	116.4	104.6	86.6	97.7	91.2	108.1	186.3	99.5	90.1	80.8	166.7	9.4	9.6	9.6	9.6	9.7	9.4	9.6	9.8	9.5
48	Male	Y	NV	37	165	51.6	18.95		Tea (two cups/ day)	P	95.1	92.9	93	94.5	101.9	87.3	87.3	94.4	106.6	84.5	48.1	56.1	66	73.8	62.8	74.1	99	117	9.6	9.7	10.2	9.6	10.5	9.9	9.5	10	9.6
49	Male	Y	NV	32	171.5	64	21.76		Tea (two cups/ day)	D2	95	107.2	96.5	88.7	98.8	95	90.2	92.8	100.6	147.7	153.4	157.3	140.8	132.9	136.1	95.5	126.1	135.2	9.2	9.3	9.5	9.5	9.9	9.7	9.5	9.6	9.9
50	Male	Y	NV	36	152	62.5	27.05		Tea (one cup/ day)	D3	83.4	87.5	83	89	83.9	82.1	141.1	87.4	82.3	144.7	95.7	140.7	147.2	196.9	129.6	181	126.8	87.7	9.1	9	9.1	9.2	9.5	9.2	9.3	9.4	9.5
51	Male	Y	NV	31	162.5	70.2	26.58			P	100.4	97.1	96.8	108.7	114.3	102.6	103.7	94.3	117.3	141.7	103.7	113.1	169	141.1	125.9	118.3	163	210.6	9.6	9.2	9.5	9.8	9.8	9.5	9.2	10.1	9.5
52	Male	Y	V	28	163.5	64.3	24.05		Tea (one cup/ day); Non tobacco pan masala; (left since 7 years)	D1	132.5	123.5	98.1	98.5	97.2	113.3	133.8	101	144.3	92.8	130.6	149.9	94.4	126.3	168	170.4	185.4	134.9	9.9	9.7	10.3	10.2	10.2	10.1	10	10.4	10.1
53	Female	Y	NV	38	138	45.5	23.89	Pre	Tea (two cups/ day)	D2	101.4	119	101.1	95.9	117.2	93.8	81.4	92.5	118.7	42.9	49.6	65	35.3	62.1	59.4	38.2	45.6	59.2	9.4	9.2	9.6	9.2	9.5	9.2	9.9	9.6	9.8
54	Female	Y	NV	25	155	60.7	25.27	Pre	Tea (two cups/ day)	P	89.1	87.7	85.3	85.2	108.9	88.1	91.1	100.5	75.6	50.6	68.8	48.5	56.6	72.1	44.9	68.7	73	102.7	9.6	10.2	9.4	9.8	9.3	9.2	9.3	9.5	9.7
55	Female	Y	NV	29	144	52.7	25.41	Pre	Tea (one cup/ day)	D3	108.1	109.6	100.5	93.2	90.6	88.3	81.8	106.7	103.2	149.1	247.4	134.5	146.6	139.1	197.5	133.6	144.5	191.1	8.9	9.4	9.1	9.2	9.4	8.9	9.4	9.4	9.6
56	Female	Y	V	33	152	63.5	27.48	Pre	Tea (one cup/ day)	D1	93.9	97.8	106.3	101.8	97.3	87.5	132.2	123.9	115.8	137.3	79.2	117	127.9	193.5	92.2	164.1	89.7	136.6	9.4	9.6	9.6	9.6	9.6	9.2	9.8	10.1	9.7
58	Female	Y	NV	43	149	55.3	24.91	Pre		D3	96.4	153.6	108.6	119.7	130.7	101.4	133.4	127	103.8	159.3	173.4	236.1	174.5	189.8	132.1	198.7	182	126.2	8.5	8.7	8.8	8.7	8.7	8.7	9	8.9	9.8
59	Female	Y	NV	44	154	71	29.94	Pre	Tea (one cup/ day)	D1	111.9	105.1	94	111.8	100.1	87.8	107.1	97.6	105	176.6	226.6	233.1	171.9	250	187.3	142	144.4	158	9.1	9.3	9.2	8.9	9.8	9.5	9.3	9.4	10
61	Male	Y	V	39	160	54.6	21.33			D1	89.4	97.3	107.2	89.3	101.9	100.1	98	128	115.3	138.1	219.9	239.2	242.4	242.6	244.5	352.4	309	238	9.1	9.2	9.5	9.8	9.4	8.9	9.6	9.5	9.8
62	Male	Y	NV	44	166	72	26.13			P	89.2	104.2	93.8	76.9	68.2	77.2	98.3	90.8	96.6	382.9	138.8	236.6	154.6	195.2	164.6	197.5	300.1	162.1	9.2	9.4	9.3	9.3	8.9	9.3	9.2	9.6	9.4
63	Male	Y	NV	31	169	76	26.61			D2	116.6	99.3	98.8	82.1	88.6	95.6	89.4	116.3	102.9	169.1	255.4	209.9	345.1	190	239.4	166.9	178.7	121.7	9.6	10.3	9.7	9.5	10.1	9.9	10.2	10.1	10.2
64	Male	Y	NV	26	164	64.5	23.98			D3	126.1	82.1	104	97.9	91.2	86.5	95.1	133.1	102.9	101.2	88.5	113.3	87.5	55	99.9	45.9	79.4	58.3	8.8	9.4	9.1	9.6	9.5	9.4	9	9.7	9.1
65	Male	Y	NV	37	154.5	46.8	19.61			D1	93.3	96.7	82.6	83	87.3	77.3	84.5	107	97.1	100.6	89	63.9	76.7	65.7	74.4	87.7	86.2	67.4	9.6	9.8	9.7	9.7	9.6	9.9	10	10.2	9.9
66	Male	Y	Veg/NV	41	170	54	18.69			D2	89.7	98.8	95.8	89.7	91.9	81.1	89.3	110.8	98	84.5	173.3	122.1	100.4	180.7	124.9	115.2	187.8	184.4	9.2	9.1	9.2	9.4	9.4	9.4	9.7	9.3	9.6
67	Male	Y	NV	34	158	58.6	23.47			D3	82.3	98.3	74.4	83.1	89.3	82.6	77.3	134.1	93.3	116.2	93.7	113.2	89.2	87.4	109.6	124.9	174.2	122.7	9.4	9.4	9.7	9.3	9.1	9.2	9.3	9.6	9.5
68	Male	Y	NV	31	163	54	20.32		Non tobacco pan masala; (left 6 years ago)	P	96.3	91.8	86.6	96.8	82.4	95.1	86.8	88	97.5	53.1	38.4	64.6	95.4	55.6	78	89.2	62.4	68.3	9.5	9.4	9.4	9.5	9.9	9.1	9.5	9.8	9.5
69	Male	Y	V	30	171	77	26.33			P	124.6	81.1	108.8	83.7	84.8	88.6	97.5	93.6	93.8	104.7	94.1	121.5	110.4	43.3	81.8	163.5	62.2	139.2	9.9	9.7	10.1	9.7	9.5	10.3	10.2	10.4	10.2
70	Male	Y	NV	34	157	52.8	21.42			D2	88.7	97.6	81.6	82.8	90.9	87.4	78.1	88.1	74.3	112.8	63.6	70.2	43.4	67.5	117.6	79	47.1	124.6	9.3	9.6	9.4	10.3	9.5	9.2	9.5	9.8	10
71	Female	Y	V	38	149	45.8	20.63	Pre	Tea (two cups/ day)	D3	95.5	110.9	85.8	81.8	93.6	85.6	94.6	97.9	97.9	101.9	128	127.3	122.8	156.9	110.2	114	128.1	100	9.4	9.5	9.5	9.4	9.6	9.5	9.7	9.9	10.3
72	Female	Y	V	38	148	46.5	21.23	Pre	Tea (two cups/ day)	D1	97.2	97.5	85.4	84	89	88.2	98.8	102.2	100.5	111.6	146.7	107.6	81	81.8	90.3	112.8	141.4	175.8	9.7	9.3	10.1	9.7	10.2	10.2	9.8	9.9	9.8
73	Female	Y	NV	26	156	64.2	26.38	Pre	Tea (two cups/ day)	P	92.5	78.1	79.1	80.2	72.9	78.2	84.4	85.4	85.3	108.1	121.9	93.4	82.3	89.4	76.4	112	88.6	76.7	10.2	9.4	9.8	9.8	9.7	9.9	9.8	9.7	10.4
74	Female	Y	NV	38	143	48.5	23.72	Pre	Tea (two cups/ day)	D3	111.6	124.6	126.4	144.3	86.9	99.3	114.8	129.5	135.4	95.2	77.9	75.9	53.3	155.9	97.9	111.9	136.7	158.5	9.7	9.5	9.6	9.1	9.8	9.5	9.6	9.6	10.4
75	Female	Y	NV	35	150.5	67	29.58	Pre	Tea (two cups/ day)	D2	98.8	122.1	110.9	113.3	95.3	87.1	103.8	121	102.2	48.5	60	100.3	61.1	101.9	50	46.2	52.3	50.1	8.9	8.9	8.9	9.2	9.5	9.1	9.3	8.9	9.2
76	Female	Y	V	27	146.5	54.5	25.39	Pre	Tea (two cups/ day)	D1	80.9	98.6	86.9	85.9	79.6	83.1	85.8	95.3	114.3	147.1	105.9	153.1	95.9	242.8	137.2	150	108.1	162.2	9.9	9.5	9.7	9.5	9.9	10.1	9.7	10.1	9.7
77	Female	Y	NV	30	144	48.5	23.39	Pre	Tea (two cups/ day)	D2	80.5	92.6	81.7	76.8	86.6	86	89.3	86.2	83.9	226.2	316.5	238.6	243.1	152.1	236.8	230.5	179.8	242.4	9.5	9.1	9.8	9.5	10.3	9.7	9.6	9.7	9.3
79	Female	Y	NV	44	147	48.5	22.44	Pre	Tea (two cups/ day)	D3	82.6	90.8	79.4	89.9	88.2	83.2	90.3	79.6	93.7	98.9	85.8	128.7	114	89.2	104.8	95.5	114.3	115.1	9	9.3	9.3	9.2	9.5	9.2	9.6	10	10.1
80	Male	Y	NV	31	165	56.7	20.83		Tea (one cup/ day)	P	82	100.6	94.8	118.1	83.1	105.9	85.7	91.8	96.2	136.7	201.7	135.2	118.4	164.2	132.5	148.6	92.7	133.1	9.5	9.4	9.5	9.2	9.8	9.3	9.5	9.4	9.9

Supplementary Table S2: A summary indicating read-number statistics in groups of samples collected at various time-points of the study

Visit	No. of samples	Average Number of Raw Sequences	Minimum Raw Sequence	Median No. of Processed Sequences
1	80	1194673	250322	153063
2	80	1050670	236786	175277
3	79	1223977	16558	218000
4	78	1050100	11262	165587
5	77	878520	13546	129328
6	76	1384794	2672	184917
7	76	1244200	35528	202330
8	73	1222842	156706	180359
9	74	1046051	18790	99297

Supplementary Table S3: A cross-comparison of most abundant OTUs in the microbiome profiles grouped as per study phases and dosage type
A matrix representation of the names of the most abundant OTUs within the microbiome profiles grouped according to study phase and dosage type.

Top 10 Microbes	Basal	Dosage	Followup
P	Prevotella.copri<br>Faecalibacterium.prausnitzii<br>Prevotella.stercorea<br>Prevotella.unclassified<br>Sutterella.OTU.8<br>Lactobacillus.ruminis.OTU.1<br>Roseburia.faecis<br>Sutterella.unclassified<br>Oscillospira.unclassified<br>Haemophilus.parainfluenzae.OTU.47	Prevotella.copri<br>Prevotella.copri.OTU.109<br>Prevotella.copri.OTU.102<br>Faecalibacterium.prausnitzii.OTU.222<br>Faecalibacterium.prausnitzii<br>Lactobacillus.ruminis.OTU.1<br>Prevotella.OTU.17<br>Prevotella.copri.OTU.106<br>Prevotella.copri.OTU.110<br>Bifidobacterium.adolescentis.OTU.4	Prevotella.copri<br>Faecalibacterium.prausnitzii<br>Lactobacillus.ruminis.OTU.1<br>Prevotella.unclassified<br>Prevotella.stercorea<br>Sutterella.OTU.8<br>Oscillospira.unclassified<br>Roseburia.faecis<br>Sutterella.unclassified<br>Haemophilus.parainfluenzae.OTU.47
D1	Prevotella.copri<br>Faecalibacterium.prausnitzii<br>Lactobacillus.ruminis.OTU.1<br>Prevotella.stercorea<br>Dialister.OTU.12<br>Oscillospira.unclassified<br>Prevotella.unclassified<br>Sutterella.OTU.8<br>Sutterella.unclassified<br>Roseburia.faecis	Prevotella.copri.OTU.109<br>Prevotella.copri<br>Faecalibacterium.prausnitzii<br>Prevotella.copri.OTU.102<br>Lactobacillus.ruminis.OTU.1<br>Dialister.OTU.12<br>Prevotella.copri.OTU.110<br>Prevotella.OTU.17<br>Sutterella.OTU.8<br>Prevotella.unclassified	Prevotella.copri<br>Faecalibacterium.prausnitzii<br>Prevotella.unclassified<br>Megasphaera.OTU.4<br>Prevotella.stercorea<br>Dialister.OTU.12<br>Lactobacillus.ruminis.OTU.1<br>Oscillospira.unclassified<br>Sutterella.OTU.8<br>Sutterella.unclassified
D2	Prevotella.copri<br>Faecalibacterium.prausnitzii<br>Prevotella.stercorea<br>Sutterella.OTU.8<br>Roseburia.faecis<br>Prevotella.unclassified<br>Sutterella.unclassified<br>Lactobacillus.ruminis.OTU.1<br>Haemophilus.parainfluenzae.OTU.47<br>Oscillospira.unclassified	Prevotella.copri<br>Prevotella.copri.OTU.109<br>Faecalibacterium.prausnitzii<br>Prevotella.copri.OTU.102<br>Lactobacillus.ruminis.OTU.1<br>Prevotella.copri.OTU.106<br>Sutterella.OTU.8<br>Prevotella.copri.OTU.110<br>Prevotella.OTU.17<br>Roseburia.faecis	Prevotella.copri<br>Faecalibacterium.prausnitzii<br>Prevotella.stercorea<br>Lactobacillus.ruminis.OTU.1<br>Prevotella.unclassified<br>Oscillospira.unclassified<br>Sutterella.OTU.8<br>Haemophilus.parainfluenzae.OTU.47<br>Dialister.OTU.12<br>Sutterella.unclassified
D3	Prevotella.copri<br>Faecalibacterium.prausnitzii<br>Prevotella.stercorea<br>Prevotella.unclassified<br>Roseburia.faecis<br>Lactobacillus.ruminis.OTU.1<br>Sutterella.unclassified<br>Dialister.OTU.12<br>Oscillospira.unclassified<br>Haemophilus.parainfluenzae.OTU.47	Prevotella.copri.OTU.109<br>Prevotella.copri<br>Prevotella.copri.OTU.102<br>Prevotella.copri.OTU.106<br>Prevotella.copri.OTU.108<br>Prevotella.OTU.17<br>Faecalibacterium.prausnitzii<br>Lactobacillus.ruminis.OTU.1<br>Prevotella.copri.OTU.110<br>Sutterella.OTU.8	Prevotella.copri<br>Faecalibacterium.prausnitzii<br>Prevotella.stercorea<br>Prevotella.unclassified<br>Dialister.OTU.12<br>Lactobacillus.ruminis.OTU.1<br>Sutterella.OTU.8<br>Megasphaera.OTU.4<br>Oscillospira.unclassified<br>Sutterella.OTU.5

Supplementary File S1: A sample template of the consent form which was used in the present study. The 17 pages comprising the entire contents of the consent form are provided from the next page.

PROJECT NO.: 14-VIN-413

INFORMED CONSENT DOCUMENT

STUDY TITLE: A randomized, double blind, placebo controlled, dose-response relationship study to investigate the efficacy of Fructo-oligosaccharides (FOS) on human gut microflora following oral doses of fructo-oligosaccharides of TATA Chemicals Ltd., India in healthy, adult, human subjects.

Version No.: 01 Registration No.:____________

Date: 23 Dec 2014

INFORMED CONSENT

- We would like to invite you to take part in a clinical research study sponsored by TATA Chemicals Ltd., India to investigate the efficacy of Fructo-oligosaccharides (FOS) on human gut microflora (microorganism species that live in the digestive tracts) following oral doses of fructo-oligosaccharides of TATA Chemicals Ltd., India.
- You are informed that video and audio recording of the informed consent process will be performed.
- Before you agree to join in this study, you need to know the risks and benefits so you can make an informed decision. This is known as "Informed Consent". This document is prepared to provide you all relevant information pertaining to your participation as a subject in this study
- You must carefully consider the implications of your participation before giving your written consent. Please read the information carefully and discuss it with anyone you want. This may include a friend or a relative or Veeda's employees. If you have questions please ask the study doctor or study staff to answer them.
- Giving your consent confirms that you are willing to participate and you have been given sufficient details about the study, and the opportunity to ask questions and enough time to consider your decision.
- Once you know about the study and the tests that will be done, you will be asked to sign this form to join this study. Your decision to take part in this study is voluntary. That means you are free to decide to join this study or not join this study. You are also free to leave the study at any time. If you choose not to join in this study, you can discuss regular medical care with the study doctor.
- The Study Doctor may remove you from this study for any reason. Any new information about the study medicine will be given to you so you may decide to continue in the study or leave it.

  You may be taken out of the study if:

  1. Staying in the study would be harmful.
  2. You need treatment not allowed in this study.
  3. You fail to follow instructions.
  4. You become pregnant (for female subjects).
  5. The study is cancelled.

You decide to leave the study you can tell the study doctor or study staff. They will make sure that proper procedures are followed and a final visit is made for your safety.

Approved By
ANVESHHAN-IEC
02 JAN 2015

REPORT COPY
Veeda Clinical Research

TRIAL PURPOSE

There are mainly two objectives to conduct this study

Primary objective:

- To determine the bifidogenic properties (Promoting the growth of beneficial bifidobacteria in the intestinal tract) of Fructo-oligosaccharides (FOS) administered at different dose level of 2.5 g/d, 5.0 g/d and 10 g/d in the diet and dose response relationship of the Fructo-oligosaccharides (FOS) at doses ranging from 2.5 to 10 g/d in comparison with a placebo controlled treatment arm in healthy, adult, human subjects.

Secondary objective:

- To measure the effect of Fructo-oligosaccharides on random blood sugar, calcium and triglycerides (fatty substance found in human blood) parameters in healthy, adult, human subjects.

Placebo drug is an inactive substance or preparation used as a control in an experiment to determine the effectiveness of a medicinal drug

TATA Chemicals Ltd., India is our client and is sponsoring this study.

The Fructo-oligosaccharides powder formulation of TATA Chemicals Ltd., India is called the Test formulation.

The Powder containing Maltodextrin of TATA Chemicals Ltd., India is called the Placebo treatment.

Name of finished products:

Test treatment 01 (T1): Fructo-oligosaccharides powder at a dose of 2.5 g/d of TATA Chemicals Ltd., India

Test treatment 02 (T2): Fructo-oligosaccharides powder at a dose of 5.0 g/d of TATA Chemicals Ltd., India

Test treatment 03 (T3): Fructo-oligosaccharides powder at a dose of 10.0 g/d of TATA Chemicals Ltd. India

Placebo treatment (P): Powder containing Maltodextrin at a dose of 10.0 g/d of TATA Chemicals Ltd., India

These experimental studies are done in healthy, adult, human subjects.

TRIAL CONDUCT

Here we describe the exact schedule of what will take place during the study and what your responsibilities are. If you agree to take part in the study, you are agreeing to adhere to this schedule. Please read it carefully and get any doubts clarified.

Approved By
ANVESHHAN-IEC
02 JAN 2015

REPORT COPY
Veeda Clinical Research

When you participate in this study as a subject, you are required to visit our facility a total of at least 12 times.

You are one of the 80 (40 males and 40 females) healthy, adult, human subjects being asked to participate in this study. Additional 10 (05 males and 05 females) subjects may be enrolled in each group of study

Approximate duration of this study will be of at least 7 months from the day of admission of study till the end of study period.

The study is comprised of 01 (one) period.

You will be informed in a timely manner, if important new information becomes available that may be relevant to your willingness to continue participation in the study.

STUDY PERIOD

Your first visit will be for getting admitted into the facility on the day of admission for the study period for informed consent presentation and obtaining written consent.

After you report at the clinical facility, you will be explained this informed consent document (ICD) in detail so that you understand all the aspects pertaining to your participation in this study. You can clarify any doubt or anything that is not clear to you from the principal investigator or someone designated by him. After you understand this ICD, if you agree to participate in this study, you will be required to sign this document. Then you will be admitted to the clinical facility. We will give you a photocopy of the signed ICD.

The study will comprise of 3 phases:

1. **Basal phases (Day 01 to 60):**

   In this phase, you will take your normal routine diet. In the first two months, stool and blood samples will be collected from you at three time point.

   The 80 (40 males and 40 females) healthy, adult, human subjects who complete this phase of study will be asked to participate in this study

   If there is no withdrawal prior to next dosage phase of study, additional 10 subjects will be discontinued after completion of basal phase of study.

2. **Dosage phase (Day 61 to 150):**

   This phase will span for 3 months in which you need to take Fructo-oligosaccharides supplement in your daily diet. Fructo-oligosaccharides powder supplement will be provided to you who completed the basal phase successfully and eligible for dosage phase. There will be 4 groups (3 - TATA

Approved By
ANVESHHAN-IEC
02 JAN 2015

REPORT COPY
Veeda Clinical Research

Chemicals Ltd., India's Fructo-oligosaccharides sample and one Placebo) for the study. There will be 4 stool and blood sample point collection during this phase.

3. **Follow-up phase (Day 151 to 210):**

The Fructo-oligosaccharides supplement will be discontinued in this phase and you will have normal diet. There will be 3 stool and blood sample collection points during this phase. This phase will span for 2 months.

Alcohol breath test will be performed on the day of each visit of sample collection. Your urine sample will be taken and screen for drugs of abuse will be performed on the day of each visit of sample collection. Urine pregnancy test (only for female) will be performed on the day of each visit of sample collection.

On admission day, we will give you a number tag, which you have to keep with you throughout your study participation. You will be given pouches of test formulation or placebo formulation of investigational products (allocated as per the randomization schedule) and subject diary.

The pouches of test formulation or placebo formulation of investigational products will be given as follow:

- We will give you 15 pouches + 05 extra pouches (if available) on day 60 visit for day 61 to day 75 dosing.
- We will give you 15 pouches on day 75 visit for day 76 to day 90 dosing.
- We will give you 30 pouches on day 90 visit for day 91 to day 120 dosing.
- We will give you 30 pouches on day 120 visit for day 121 to day 150 dosing.

You are requested to bring your diary and used/unused pouches during your visit at clinical facility of Veeda Clinical Research Pvt. Ltd. Extra pouches will be given to for loss of any dosing pouch or any other reason.

Mode of administration of investigational products:

You will have to consume a daily dose of Fructo-oligosaccharides powder of 2.5 g or 5.0 g or 10.0 g or 10.0 g of placebo (allocated as per the randomization schedule) from day 61 to day 150 after dissolved in approximately 240 ml of drinking water at ambient temperature and to be taken at home after dinner.

You will have to make entry of the same in diary allotted to you with date and time.

This is a double-blind study. Persons involved in this study (i.e. investigator, physicians/nurses, study monitor and analytical personnel) and you will remain blinded at all times about the investigational product treatment, unless in the case of an emergency if it is required.

Approved By
ANVESHHAN-IEC
02 JAN 2015

REPORT COPY
Veeda Clinical Research

Stool and blood sampling schedule:

You will have to report to clinical facility for 1, 30, 60, 75, 90, 120, 150, 165, 180 and 210 days ambulatory stool and blood samples in study.

A total of ten (10) stool samples will be collected during the study over a period of 7 months from each subject.

A total of ten (10) blood samples will be collected during the study over a period of 7 months from each subject.

Stool and blood samples will be collected preferably in the morning of scheduled visit at Veeda Clinical Research Pvt. Ltd. You will be instructed to visit clinical facility for sample.

1. **Basal phases:**

   In the first two months (60 days), three stool samples will be collected from you at three time points on day 1, 30 and 60.

   4.0 mL blood samples will be collected from you at three time point on day 1, 30 and 60.

2. **Dosage phase:**

   Four stool samples will be collected from the subject at four time point on day 75, 90, 120 and 150.

   4.0 mL blood samples will be collected from the subject at four time point on day 75, 90, 120 and 150.

3. **Follow-up phase:**

   Three stool samples will be collected from the subject at three time point on day 165, 180 and 210.

   4.0 mL blood samples will be collected from the subject at three time point on day 165, 180 and 210.

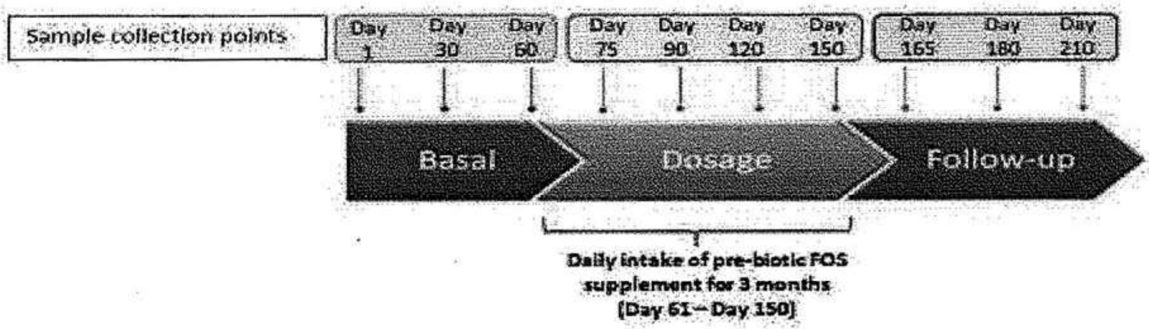

Approved By ANVESHHAN-IEC 02 JAN 2015

REPORT COPY
Veeda Clinical Research

Stool sample collection:

1. You will visit at clinical facility of Veeda Clinical Research Pvt. Ltd
2. You will be instructed to empty your bladder before beginning the collection.
3. Fecal sample free of urine or toilet water will be collected in suitable container.
4. If sample is liquid or diarrhea, you will have to wait until the next bowel movement to collect samples.
5. Toilet papers or tissues will be provided.

Blood sample collection:

Blood samples will be collected at scheduled blood sample collection day visit at Veeda Clinical Research Pvt. Ltd through direct vein puncture from forearm vein.

Stool volume:

Sufficient volume will be separated for sample from total sample.

Blood volume:

For each subject, a total of 10 blood samples will be collected in the study. The total blood volume will not exceed 52.0 mL for male and female subject as follows:		
For pharmacodynamic analysis	:	40.0 mL
For screening	:	08.0 mL
For post study safety assessment	:	04.0 mL
In addition to above up to additional of 05.0 mL blood sample will be collected if required, for hemolyzed sample or clotted sample or sample loss or any other reason. Follow-up may require additional blood samples to be drawn.		

If you wish to participate in this study as a subject, you have to agree to donate the required quantity of stool as per the sampling times.

You will have to agree to donate the necessary quantity of blood, as mentioned above, if you wish to be included as a subject for the study. For your information, our body is provided with such a system that this much quantity of blood is re-formed within a few weeks only.

Clinical examination (vital signs (sitting blood pressure, oral body temperature, radial pulse rate and respiratory rate), physical examination and systemic examination) will be done on day 1, 30, 60, 75, 90, 120, 150, 165, 180 and 210 (after collection of last sample) of the study.

Clinical examination may also be done at any time during the conduct of the study, if the Clinical Research Physician feels it necessary..

Approved By
ANVESHHAN-IEC
02 JAN 2015

REPORT COPY
Veeda Clinical Research

Subjects will be questioned for well being at the time of clinical examination and recording of sitting blood pressure and radial pulse rate and on the day of collection of the stool and blood sample.

Post study safety assessment (Hemoglobin, Total Count, Differential Count, Platelet count and Biochemical parameters - SGOT (Serum Glutamate Oxaloacetate Transaminase - enzyme that is normally present in liver) and SGPT (Serum Glutamate Pyruvate Transaminase- enzyme that is normally present in liver) Bilirubin, Creatinine and Urea) will be done at the end of study.

BACKGROUND INFORMATION

Below, we will describe the background information about fructo-oligosaccharides, which are being studied here and other relevant information. Please read this information and clarify if you have any queries before you decide to participate in this study as a subject.

Therapeutic Uses:

Fructo-oligosaccharides has prebiotics (chemicals that induce the growth and/or activity of commensal microorganisms (e.g., bacteria and fungi) that contribute to the well-being) properties. It is non-digestible food ingredient that beneficially affects the host by selectively stimulating the growth and/or the activity of one or a limited number of bacterial species in the colon.

Fructo-oligosaccharides might improve cholesterol (fatty substance found in human blood) profiles by 5%, an amount too small to make much of a difference in most circumstances.

Fructo-oligosaccharides has also been suggested for preventing traveler's diarrhea (occurrence of multiple, loose, watery stool in someone traveling to an area outside their usual surroundings). However, in a large (244-participant) double-blind study, Fructo-oligosaccharides at a dose of 10 g daily again offered only minimal benefits. 8 Probiotics (organisms such as bacteria or yeast that are believed to improve health) themselves might be a better bet. Another study found that use of Fructo-oligosaccharides might help reduce incidents of diarrhea (frequent and watery passage of stool), flatulence (excessive formation of gases in the stomach or intestine), and vomiting in preschoolers.

Fructo-oligosaccharides have been advocated as a treatment for irritable bowel syndrome (disorder with abdominal pain, diarrhea or constipation).

Small double-blind studies found that Fructo-oligosaccharides at a dose of 10 g daily may improve magnesium absorption in postmenopausal women. Whether this is beneficial remains unclear, since magnesium deficiency is not believed to be a widespread problem. Fructo-oligosaccharides may also slightly increase copper absorption, but does not appear to affect absorption of calcium, zinc, or selenium.

A randomized, placebo-controlled trial, involving 134 infants less than 6 months old whose parents suffered from allergies, found that those fed a prebiotic (chemicals that induce the growth and/or activity of commensal microorganisms (e.g., bacteria and fungi) that contribute to the well-being) combination of FOS (Fructo-oligosaccharides)/GOS (Galactooligosaccharides) experienced a significant reduction in both allergy symptoms and minor infections that lasted at least through age 2. The researchers suggested that the

Approved By
ANVESHHAN-IEC
02 JAN 2015

REPORT COPY
Veeda Clinical Research

favorable effects of prebiotics (chemicals that induce the growth and/or activity of commensal microorganisms (e.g., bacteria and fungi) that contribute to the well-being) on intestinal bacteria early in life may produce lasting benefits to the immune system.

Dosage and Administration:

When taken simply for promoting healthy bacteria, Fructo-oligosaccharides are often taken at a dose of 4-6 g daily. When used for therapeutic purposes, the typical dose of Fructo-oligosaccharides is 10-20 g daily, divided into three doses and taken with meals. Side effects are common at a daily intake 15 g or more.

Adverse Reactions:

Ingesting doses that are too large (which varies from individual to individual) can cause increased colonic fermentation (The breakdown of dietary fibre, starch, and some other undigested foods by bacteria in the large intestine) that can result in flatulence (excessive formation of gases in the stomach or intestine) and/or diarrhea (frequent and watery passage of stool).

Fructo-oligosaccharides appear to be generally safe. However, they can cause bloating (increase in diameter of the abdominal area), flatulence (excessive formation of gases in the stomach or intestine), and intestinal discomfort, especially when taken at doses of 15 g or higher daily. People with lactose intolerance (body cannot easily digest lactose, a type of natural sugar found in milk and dairy products) may particularly suffer from these side effects

DOSE FOR THE SUBJECT IN THIS STUDY

Fructo-oligosaccharides powder of 2.5 g or 5.0 g or 10.0 g or 10.0 g of placebo will have to take with approximately 240mL drinking water at ambient temperature and to be taken at home after dinner in study.

RESTRICTIONS TO BE FOLLOWED

If you participate in this project as a subject, you will be required to follow the following restrictions:

Food and water:

You will be instructed to exclude fermented dairy products like yogurt containing viable bifidobacteria from their diet and to limit consumption of foods containing high levels of nondigestible oligosaccharides such as onion, asparagus, wheat, rye.

Medications:

You will have to refrain from taking any medication prescribed to you within 1 and over-the-counter medication including antibiotics last two weeks prior to day 01 of basal phase till completion of the study. This is because those medicines may interact with the drug under study and may cause health problems apart from affecting the bioavailability profile of the drug under study. If you take any medication during this period, please inform to the Principal Investigator or any study personnel and also inform your physician that you are participating in a drug trial for the study drug (you can ask your physician to contact the Principal Investigator for any further details).

Approved By
ANVESHHAN-IEC
02 JAN 2015

REPORT COPY
Veeda Clinical Research

Others:

You will be instructed to refrain from smoking, chewing tobacco, pan or pan masala, gutkha, masala (containing betel nut and tobacco) and from consuming any alcohol or alcoholic products, grapefruit juice, xanthine-containing foods or beverages (like chocolate or cola drinks) from 48.00 hours prior to basal phase of study till the completion of the study.

BIRTH CONTROL, DANGERS OF PREGNANCY AND BREASTFEEDING

For Female subjects: You should not be in the study if, you are pregnant or are breastfeeding. You should not have unprotected sexual intercourse with any non-sterile male partner (i.e. male who has not been sterilized by vasectomy for at least 6 month) right from basal phase of study till the end of study. Moreover, you will be advised to use an acceptable method of birth control such as condoms, foams, jellies, diaphragm, intrauterine device (IUD), or abstinence during study duration. If you are pregnant or become pregnant during the study, the study drug or procedures may involve risks to the unborn baby, which are currently unforeseeable. If you become pregnant during the study, you must be instructed to inform the study doctor immediately. It is not known whether the study drug is safe for breast fed babies. Therefore if you are breastfeeding a child then it may not be safe for them to participate in the study.

WITHDRAWAL

- You are free to withdraw yourself from the study at any time during the course of the study without providing any reason thereof.
- You may be asked to withdraw from the study if you fail to abide by the restrictions to be followed or if you cease to be physically and/or clinically fit, on examination, for further participation in the study. In either of the cases, you will not be entitled for any penalty or you will not lose your right for the study-related medical care and your right for voluntary participation in future studies. You will be paid the participation fee as the IEC (Independent Ethics Committee) specifies for the particular case.
- The Sponsor and the Principal Investigator reserve the right to discontinue the study for safety reasons at any time.

Benefits of treatment

You will have no direct benefit from participating in this research study, except that you will have a complete health examination. You will incur no expenses for participation in this study. However your participation may help development of new drugs to improve cholesterol (fatty substance found in human blood) profiles, for preventing traveler's diarrhea (occurrence of multiple, loose, watery stool in someone traveling to an area outside their usual surroundings), help reduce incidents of diarrhea (frequent and watery passage of stool), flatulence (excessive formation of gases in the stomach or intestine), and vomiting in preschoolers.

Alternative treatment

Since this study is only for research purpose on healthy volunteers, the only alternative would be not to participate in the study. Your participation in the study is at your own free will, and you will give your consent only after you have understood details of the study.

Approved By
ANVESHHAN-IEC
02 JAN 2015

REPORT COPY
Veeda Clinical Research

ETHICAL PROCEDURES

Since there are many technical details about how this study is done, we submit our protocols to an independent panel of eminent personalities for their review and approval. Some of these people are doctors, some are pharmacists, some are judges, some are social workers and some are religious leaders. All members on this panel (called an Independent Ethics Committee or IEC) are not associated with Veeda clinical research Pvt. Ltd. in any way and hence they are independent and free to criticize any aspect of the study. This panel has examined our current study from many angles: Is it reasonably safe for subjects? Are all the possible risks adequately explained? Is there a less risky way to do the experiment? Are the subjects being taken advantage of? Is this study necessary for proving bifidogenic properties (Promoting the growth of beneficial bifidobacteria in the intestinal tract)? and many other questions.

The entire protocol, as well as this Informed Consent Document and CRF have been scrutinized by the IEC and have been found to be acceptable. No study is ever conducted at Veeda clinical research Pvt. Ltd. without such approval. The IEC also has the authority to examine the implementation of the study and if major deviations are found, the IEC has the authority to put a stop to an ongoing study.

SAFETY PROCEDURES

1. Rigorous screening is done for all subjects to ensure that they are fit enough to participate in the study.
2. During the study because the possible adverse effects of this drug (as described above in Background Information) you are requested to contact Principal Investigator at any time and/or visit Veeda Clinical Research clinical facility.

FINANCIAL CONSIDERATIONS

1. For the time and effort you put into participating in this study, you will be paid a sum of Rs. 12000/- (Rupees twelve thousand only) proportionately at the end of each period of the study as per the break up as below:
    a. Day 01 visit: Rs. 500/-
    b. Day 30 visit: Rs. 500/-
    c. Day 60 visit: Rs. 500/-
    d. Day 75 visit: Rs. 1000/-
    e. Day 90 visit: Rs. 1000/-
    f. Day 120 visit: Rs. 1000/-
    g. Day 150 visit: Rs. 1000/-
    h. Day 165 visit: Rs. 500/-
    i. Day 180 visit: Rs. 500/-
    j. Day 210 visit: Rs. 5000/-
    k. End of post study follow up: Rs. 500/-

   If you ask for the same, Rest of the payment will be given at the end of the study.
2. If you will be enrolled as an extra subject then you will be discontinued after completion of basal phase of study, you will be paid a sum of Rs. 4000/- (Rupees four thousand only) as per the break up below:
    a. Day 01 visit: Rs. 500/-
    b. Day 30 visit: Rs. 500/-

Approved By
ANVESHHAN-IEC
02 JAN 2015

REPORT COPY
Veeda Clinical Research

   c. Day 60 visit: Rs. 3000/-
3. You will not be required to pay anything for study related expenses.
4. If you discontinue before completion of the project, you will be paid proportionate to your participation.
5. If we advise you to discontinue because of developing adverse event or on medical ground before completion of the project, full participation fee will be paid to you.
6. If we ask you to discontinue because of misconduct as a disciplinary action, the payment will be decided on a case-by-case basis.
7. In case of any disputes pertaining to partial payments, you may approach the IEC and the decision of the IEC will be binding on you as well as Veeda clinical research Pvt. Ltd.
8. In case of study related injury or death M/s. Veeda Clinical Research Pvt. Ltd., Ahmedabad will provide complete medical care along with compensation for the injury or death. We have an insurance policy to cover you in case of serious adverse events.
9. You will be provided free medical help for all adverse event(s)/serious adverse event (s) from study start to study end date. We have an insurance policy to cover you in case of AE / serious adverse events.
10. Compensation in case of injury or death during study
    Any injury or death occurring in study due to following reasons, shall be consider as study related injury or death and you or your nominee (s) as the case may be are entitled for financial compensation as per order of regulatory authorities which will be over and above expenses incurred on the medical management and shall be borne by the Veeda Clinical Research Pvt. Ltd., Ahmedabad-380015 of the study.
    1. Adverse effect of investigational product (s)
    2. Violation of the approved protocol, scientific misconduct or negligence by the sponsor or his representative or the Investigator.
    3. Failure of investigational product to provide intended therapeutic effect
    4. Use of placebo in a placebo-control trial
    5. Adverse effects due to concomitant medication excluding standard care, necessitated as part of approved protocol
    6. For injury to a child in-utero because of the participation of parent in study
    7. Any clinical trial procedures involved in the study.

    In case of study related injury or death, ethics committee and Indian regulatory authorities will decide the quantum of compensation, to be paid by Veeda Clinical Research Pvt. Ltd, to you or your nominee(s). The compensation will be paid within one month from receipt of order from regulatory authorities.

CONFIDENTIALITY

For the audio-visual recording of informed consent process, your identity and records are as far as possible kept confidential; and that no details about your identity will be disclosed without valid scientific and legal reasons which may be essential for the purposes of therapeutics or other interventions, without your specific consent in writing.

The records of your medical history, physical examination, laboratory results and any other information or data generated during this study will be reviewed by the sponsor pharmaceutical company, monitor, auditor,

Approved By
ANVESHHAN-IEC
02 JAN 2015

REPORT COPY
Veeda Clinical Research

ethics committee and inspected by Indian and foreign regulatory authorities. By signing this written informed consent form, you are authorizing direct access to the above mentioned records. A pre-condition for entry into the study is your agreement to release all of these above-mentioned documentation (which also include the medical records) and data for any lawful purpose. This data can also be shipped to another country and results may also be published. In such cases your name will be removed from all documentation to ensure anonymity. You may have access to your medical reports once the study is finished.

You have the right to cancel this consent at any time. If you cancel this consent, then Veeda clinical research Pvt. Ltd and investigator on the trial will no longer use or disclose your medical information, unless it is necessary to do so to preserve the scientific integrity of the study. However, canceling this consent will not affect previous uses and disclosures and your medical information would not be removed from the study records.

CONTACT INFORMATION

You need to keep any of your close associates (family, friends or others) informed about your possible participation in this research study at Veeda. This would help Veeda reach out to you or your close associate for possible medical and other assistance in case of emergency.

You are required to contact any of the following persons for any clarification regarding the study or any kind of discomfort, injury or adverse event faced during the conduct of the study.

For questions about our location, how to get here, and what time to report, contact	:	Mr. Hemal Shah, (O): 079-3061 1500/02;<br>(M): 98795 90830.<br>Mr. Rakesh Gadhvi, (M): 99099 30330
For questions about technical details of the study and questions on why the study is important, contact	:	Dr. Gunjan Shah,<br>Principal Investigator,<br>Phone: 079-3001 3000
To contact the IEC, for how the study was evaluated or to get a neutral opinion on this study or for any query pertaining to your rights, contact	:	**Contact:** Dr. Mira Desai, IEC Chairperson,<br>Anveshhan Independent Ethics Committee<br>**Address:** B-8, Simandhar Residency,<br>Near Gulab tower,<br>Behind Utopia school, Thaltej,<br>Ahmedabad-380054, Gujarat, INDIA<br>**Phone:** 079-64502818

Approved By
ANVESHHAN-IEC
02 JAN 2015

REPORT COPY
Veeda Clinical Research

TIME TABLE OF EVENTS

The following is a representative time schedule for one subject assuming that the study medication will be administered at 08:00 (tentative time). Timings for other subjects will be uniformly staggered:

DAY	TIME RELATIVE TO DOSING (hours)	APPROXIMATE TIME	EVENTS
-1	-24.00 to -12.00 hr	08:00-20:00	Compliance assessment, Breath test for alcohol consumption, urine pregnancy test (only for female volunteers), Urine screen for drug of abuse, Clinical examination, Reporting for Informed consent, ICD Presentation and Obtaining Written Consent (period 01 only), , Criteria Check (period 01 only) and admission in study
1	07.00 to 12.00 hr	07:00-12:00	Stool, blood sample collection, Breath test for alcohol consumption, urine pregnancy test (only for female volunteers)Urine screen for drug of abuse, Clinical examination.
30	07.00 to 12.00 hr	07:00-12:00	Stool, blood sample collection, Breath test for alcohol consumption, urine pregnancy test (only for female volunteers)Urine screen for drug of abuse, Clinical examination.
60	07.00 to 12.00 hr	07:00-12:00	Stool, blood sample collection, Breath test for alcohol consumption, urine pregnancy test (only for female volunteers)Urine screen for drug of abuse, Clinical examination.
75	07.00 to 12.00 hr	07:00-12:00	Stool, blood sample collection, Breath test for alcohol consumption, urine pregnancy test (only for female volunteers)Urine screen for drug of abuse, Clinical examination.
90	07.00 to 12.00 hr	07:00-12:00	Stool, blood sample collection, Breath test for alcohol consumption, urine pregnancy test (only for female volunteers)Urine screen for drug of abuse, Clinical examination.
120	07.00 to 12.00 hr	07:00-12:00	Stool, blood sample collection, Breath test for alcohol consumption, urine pregnancy test (only for female volunteers)Urine screen for drug of abuse, Clinical examination.
150	07.00 to 12.00 hr	07:00-12:00	Stool, blood sample collection, Breath test for alcohol consumption, urine pregnancy test (only for female volunteers), Urine screen for drug of abuse, Clinical examination.

Approved By
ANVESHHAN-IEC
02 JAN 2015

REPORT COPY
Veeda Clinical Research

DAY	TIME RELATIVE TO DOSING (hours)	APPROXIMATE TIME	EVENTS
165	07.00 to 12.00 hr	07:00-12:00	Stool, blood sample collection, Breath test for alcohol consumption, urine pregnancy test (only for female volunteers), Urine screen for drug of abuse, Clinical examination.
180	07.00 to 12.00 hr	07:00-12:00	Stool, blood sample collection, Breath test for alcohol consumption, urine pregnancy test (only for female volunteers), Urine screen for drug of abuse, Clinical examination.
210	07.00 to 12.00 hr	07:00-12:00	Stool, blood sample collection, Breath test for alcohol consumption, urine pregnancy test (only for female volunteers), Urine screen for drug of abuse, Clinical examination.

Post-study safety assessment and clinical examination will be done at end of study.

Please read the declarations mentioned below and if you feel comfortable about taking part in the study, put your signature at the specified space of this document. Please note that there is no pressure of any kind from Veeda clinical research Pvt. Ltd. on you to participate. Please take part only if you are fully satisfied that you would like to do so.

DECLARATIONS BY THE VOLUNTEER

- I have read this informed consent document, it is explained to me to my satisfaction, and I have understood it. Where I had doubts or questions, I had them clarified by study personnel.
- I understand that I am deemed medically fit enough to participate in this project and I will not gain any therapeutic benefit from participating in this study.
- I understand that I will not take up any financial encumbrance as a result of taking part in this study. All diagnostic costs and expenses related to any hospitalizations will be borne by Veeda clinical research Pvt. Ltd.
- I understand the risks to me of taking part in this study as explained in the adverse / serious adverse event section of BACKGROUND INFORMATION. I understand that these risks include possible hospitalization.
- I understand that the costs of any such hospitalization will be borne by Veeda clinical research Pvt. Ltd.
- I understand that I have to be present in Veeda clinical research Pvt. Ltd.' clinical facility as detailed in the timetable of events. I undertake to be so present and to comply with other instructions I obtain from the study personnel.
- I agree to take the investigational product as instructed by the study personnel and to donate the requisite quantity of blood and stool as explained in this document. I know that samples will be collected through fresh vein puncture at each sampling.
- I understand that this project is being done for research purposes only.

Approved By
ANVESHHAN-IEC
02 JAN 2015

REPORT COPY
Veeda Clinical Research

- I hereby allow the qualified/experienced staff and/or contract staff of Veeda clinical research Pvt. Ltd. to carry out the project related activities upon me and agree to cooperate with them.
- I hereby allow Veeda clinical research Pvt. Ltd. to shift me to a hospital in case of any emergency and to provide necessary treatments to me, if required to do so.
- I declare that I did not have blood loss excluding volume drawn at screening (more than 100 ml within 30 days; more than 200 ml within 60 days) prior to participate in this study.
- I declare that I have not donated blood (1 unit - 450 mL) in the past 90 days prior to participation in this study.
- I understand the dietary and other restrictions as mentioned in this document. I agree to abide by these dietary and other restrictions.
- I agree not to commit any misbehavior or misconduct with any study personnel or with any other fellow subject, having done any such thing will make me liable for legal consequences.
- I agree not to cause any damage or loss of any property of Veeda clinical research Pvt. Ltd.
- I am 18 years or older. I have given facts to the best of my knowledge to study personnel about my medical and family history.
- I am aware that my identity and personal details will be kept confidential and will not be revealed to anyone except the IEC, the Regulatory Agency (ies) and the Sponsor's inspectors/auditors. I allow Veeda clinical research Pvt. Ltd. to disclose my identity and personal details to these entities.
- I am aware that I can withdraw my consent from this project at any time during the course of the project even without disclosing the reason(s) thereof and that I shall not be deprived of any medical care that I should get for participation in this project and this will not take away my right for future participation in such projects.
- I am giving my consent voluntarily and absolutely free from duress of any kind.
- I am aware that a photocopy of this signed document will be provided to me for my reference.
- I am provided with the contact details of all the relevant persons whom I can contact for any project-related query or queries pertaining to my rights as a subject.
- I declare that I will not claim any commercial benefit on the product developed as a result of work carried out on biological samples collected during the study.

Approved By
ANVESHHAN-IEC
02 JAN 2015

REPORT COPY
Veeda Clinical Research

Informed Consent Signature Form

Study Number	14-VIN-413	
Version No.	01	Date: 23 Dec 2014
Volunteer's Name		
Volunteer's Signature		Age(years):
Address of the volunteer		
Qualification		Annual Income of the volunteer:
Occupation	Student/Self-Employed/Service/Housewife/Others (Please tick as appropriate)	
Nominee(s) (for the purpose of compensation in case of trial related death):		
Name and address of the nominee(s)		
Relation to the volunteer		

Sr. No.		Signature of Volunteer/LAR /Impartial witness
1	I confirm that I have read (Page no.: 01 to 17) and understood/have been explained the informed consent document dated 23 Dec 2014, Version No. 01 for the above study and have had the opportunity to ask questions, and have had these questions answered satisfactorily.	
2	I understand that my participation in the study is voluntary and that I am free to withdraw at any time, without giving any reason, without my medical care or legal rights being affected.	
3	I understand that the Sponsor of this study, others working on the Sponsor's behalf, the Ethics Committee and the regulatory authorities will not need my permission to look at my health records both in respect of the current study and any further research that may be conducted in relation to it, even if I withdraw from the trial. I agree to this access. However, I understand that my identity will not be revealed in any information released to third parties or published.	
4	I agree not to restrict the use of any data or results that arise from this study provided such a use is only for scientific purpose(s).	

Approved By
ANVESHHAN-IEC
02 JAN 2015

REPORT COPY
Veeda Clinical Research

5	I agree to take part in the above study.	
6	I may have to leave study without my consent, if I need other treatment, do not follow study plan, have a study related injury, or for any other reason.	
7	If I leave the study for any reason, the study doctor may ask for some end-of-study tests.	
8	I am aware that the entire informed consent process has been recorded (audio and video) after giving my oral consent.	

Signature (or thumb impression) of the Volunteer/ Legally Acceptable Representative (LAR):	______________________	Time: _____:______<br>Date: ____________
Signatory's Name	______________________	
Signature of Impartial Witness (If necessary):	______________________	Time: _____:______<br>Date: ____________
Name of Impartial Witness:	______________________	
Signature of the Investigator:	______________________	Date: ____________
Name of Investigator:	______________________	

I have received a signed copy of this Informed Consent Document.*	
**Signature of Subject / LAR / Impartial Witness**	

Original copy to be filed with the investigator and duplicate copy to be handed over to the subject.

Approved By
ANVESHHAN-IEC
02 JAN 2015

REPORT COPY
Veeda Clinical Research

Supplementary File S3: The 16S rRNA sequences generated in this study have been deposited into the European Nucleotide Archive with accession number PRJEB28572. The corresponding metadata information is provided in the below table.

Subject	Dosage	Day1	Accession number	Day60	Accession number	Day75	Accession number	Day 90	Accession number	Day 120	Accession number
1	T1	Visit1_20	ERR2803700	Visit2_1	ERR2803868	Visit3_1	ERR2803951	Visit4_32	ERR2804045	Visit5_1	ERR2804109
2	P	Visit1_22	ERR2803702	Visit2_2	ERR2803879	Visit3_2	ERR2803962	Visit4_31	ERR2804044	Visit5_2	ERR2804120
3	T2	Visit1_23	ERR2803703	Visit2_3	ERR2803890	Visit3_3	ERR2803973	Visit4_30	ERR2804043	Visit5_3	ERR2804131
4	T3	Visit1_10	ERR2803689	Visit2_73	ERR2803927	Visit3_4	ERR2803984	Visit4_29	ERR2804041	Visit5_4	ERR2804142
5	T3	Visit1_4	ERR2803732	Visit2_4	ERR2803901	Visit3_5	ERR2803995	Visit4_28	ERR2804040	Visit5_5	ERR2804153
6	T2	Visit1_19	ERR2803698	Visit2_5	ERR2803912	Visit3_6	ERR2804006	Visit4_27	ERR2804039	Visit5_6	ERR2804164
7	T1	Visit1_15	ERR2803694	Visit2_6	ERR2803923	Visit3_7	ERR2804017	Visit4_26	ERR2804038	Visit5_7	ERR2804173
8	P	Visit1_13	ERR2803692	Visit2_7	ERR2803934	Visit3_8	ERR2804019	Visit4_25	ERR2804037	Visit5_8	ERR2804174
9	T2	Visit1_18	ERR2803697	Visit2_8	ERR2803939	Visit3_9	ERR2804020	Visit4_24	ERR2804036	Visit5_9	ERR2804175
10	P	Visit1_17	ERR2803696	Visit2_9	ERR2803940	Visit3_10	ERR2803941	Visit4_23	ERR2804035	Visit5_10	ERR2804099
11	T3	Visit1_21	ERR2803701	Visit2_10	ERR2803858	Visit3_11	ERR2803942	Visit4_22	ERR2804034	Visit5_11	ERR2804100
12	T1	Visit1_14	ERR2803693	Visit2_11	ERR2803859	Visit3_12	ERR2803943	Visit4_21	ERR2804033	Visit5_12	ERR2804101
13	T3	Visit1_48	ERR2803730	Visit2_12	ERR2803860	Visit3_13	ERR2803944	Visit4_33	ERR2804046	Visit5_13	ERR2804102
14	T1	Visit1_53	ERR2803736	Visit2_13	ERR2803861	Visit3_14	ERR2803945	Visit4_34	ERR2804047	Visit5_14	ERR2804103
15	T2	Visit1_5	ERR2803743	Visit2_79	ERR2803933	Visit3_15	ERR2803946	Visit4_35	ERR2804048	Visit5_15	ERR2804104
16	P	Visit1_12	ERR2803691	Visit2_76	ERR2803930	Visit3_16	ERR2803947	Visit4_36	ERR2804049	Visit5_16	ERR2804105
17	T3	Visit1_49	ERR2803731	Visit2_14	ERR2803862	Visit3_17	ERR2803948	Visit4_37	ERR2804050	Visit5_17	ERR2804106
18	T1	Visit1_51	ERR2803734	Visit2_15	ERR2803863	Visit3_18	ERR2803949	Visit4_38	ERR2804051	Visit5_18	ERR2804107
19	P	Visit1_50	ERR2803733	Visit2_16	ERR2803864	Visit3_19	ERR2803950	Visit4_39	ERR2804052	Visit5_19	ERR2804108
20	T2	Visit1_52	ERR2803735	Visit2_17	ERR2803865	Visit3_20	ERR2803952	Visit4_40	ERR2804054	Visit5_20	ERR2804110
21	T1	Visit1_55	ERR2803738	Visit2_18	ERR2803866	Visit3_21	ERR2803953	Visit4_41	ERR2804055	NA	NA
22	T3	Visit1_70	ERR2803755	Visit2_19	ERR2803867	Visit3_22	ERR2803954	Visit4_42	ERR2804056	Visit5_21	ERR2804111
23	P	Visit1_54	ERR2803737	Visit2_20	ERR2803869	Visit3_23	ERR2803955	Visit4_43	ERR2804057	Visit5_22	ERR2804112
24	T2	Visit1_57	ERR2803740	Visit2_21	ERR2803870	Visit3_24	ERR2803956	Visit4_44	ERR2804058	Visit5_23	ERR2804113
25	T3	Visit1_66	ERR2803750	Visit2_22	ERR2803871	Visit3_25	ERR2803957	Visit4_45	ERR2804059	Visit5_24	ERR2804114
26	T2	Visit1_80	ERR2803766	Visit2_23	ERR2803872	Visit3_26	ERR2803958	Visit4_46	ERR2804060	Visit5_25	ERR2804115
27	T1	Visit1_61	ERR2803745	Visit2_24	ERR2803873	Visit3_27	ERR2803959	Visit4_47	ERR2804061	Visit5_26	ERR2804116
28	P	Visit1_1	ERR2803699	Visit2_77	ERR2803931	Visit3_28	ERR2803960	Visit4_48	ERR2804062	Visit5_27	ERR2804117
29	T2	Visit1_78	ERR2803763	Visit2_25	ERR2803874	Visit3_29	ERR2803961	Visit4_49	ERR2804063	Visit5_28	ERR2804118
30	T3	Visit1_87	ERR2803773	Visit2_26	ERR2803875	Visit3_30	ERR2803963	Visit4_50	ERR2804065	Visit5_29	ERR2804119

Subject	Dosage	Day1	Accession number	Day60	Accession number	Day75	Accession number	Day 90	Accession number	Day 120	Accession number
31	T1	Visit1_60	ERR2803744	Visit2_27	ERR2803876	Visit3_31	ERR2803964	Visit4_51	ERR2804066	Visit5_30	ERR2804121
32	P	Visit1_56	ERR2803739	Visit2_28	ERR2803877	Visit3_32	ERR2803965	Visit4_52	ERR2804067	Visit5_31	ERR2804122
33	T2	Visit1_58	ERR2803741	Visit2_29	ERR2803878	Visit3_33	ERR2803966	Visit4_53	ERR2804068	Visit5_32	ERR2804123
34	T3	Visit1_67	ERR2803751	Visit2_30	ERR2803880	Visit3_34	ERR2803967	Visit4_54	ERR2804069	Visit5_33	ERR2804124
35	P	Visit1_69	ERR2803753	Visit2_31	ERR2803881	Visit3_35	ERR2803968	Visit4_55	ERR2804070	Visit5_34	ERR2804125
36	T1	Visit1_68	ERR2803752	Visit2_32	ERR2803882	Visit3_36	ERR2803969	Visit4_56	ERR2804071	Visit5_35	ERR2804126
37	T3	Visit1_59	ERR2803742	Visit2_33	ERR2803883	Visit3_37	ERR2803970	Visit4_57	ERR2804072	Visit5_36	ERR2804127
38	P	Visit1_64	ERR2803748	Visit2_34	ERR2803884	Visit3_38	ERR2803971	Visit4_58	ERR2804073	Visit5_37	ERR2804128
39	T2	Visit1_77	ERR2803762	Visit2_35	ERR2803885	Visit3_39	ERR2803972	Visit4_59	ERR2804074	Visit5_38	ERR2804129
40	T1	Visit1_79	ERR2803764	Visit2_36	ERR2803886	Visit3_40	ERR2803974	Visit4_60	ERR2804076	Visit5_39	ERR2804130
41	T1	Visit1_26	ERR2803706	Visit2_37	ERR2803887	Visit3_41	ERR2803975	Visit4_61	ERR2804077	Visit5_40	ERR2804132
42	P	Visit1_33	ERR2803714	Visit2_38	ERR2803888	Visit3_42	ERR2803976	Visit4_62	ERR2804078	Visit5_41	ERR2804133
43	T3	Visit1_71	ERR2803756	Visit2_39	ERR2803889	Visit3_43	ERR2803977	Visit4_63	ERR2804079	Visit5_42	ERR2804134
44	T2	Visit1_37	ERR2803718	Visit2_40	ERR2803891	Visit3_44	ERR2803978	Visit4_64	ERR2804080	Visit5_43	ERR2804135
45	T1	Visit1_41	ERR2803723	Visit2_41	ERR2803892	Visit3_45	ERR2803979	Visit4_65	ERR2804081	Visit5_44	ERR2804136
46	T3	Visit1_39	ERR2803720	Visit2_42	ERR2803893	Visit3_46	ERR2803980	Visit4_66	ERR2804082	Visit5_45	ERR2804137
47	T2	Visit1_72	ERR2803757	Visit2_43	ERR2803894	Visit3_47	ERR2803981	Visit4_67	ERR2804083	Visit5_46	ERR2804138
48	P	Visit1_73	ERR2803758	Visit2_44	ERR2803895	Visit3_48	ERR2803982	Visit4_68	ERR2804084	Visit5_47	ERR2804139
49	T2	Visit1_74	ERR2803759	Visit2_45	ERR2803896	Visit3_49	ERR2803983	Visit4_69	ERR2804085	Visit5_48	ERR2804140
50	T3	Visit1_75	ERR2803760	Visit2_46	ERR2803897	Visit3_50	ERR2803985	Visit4_70	ERR2804087	Visit5_49	ERR2804141
51	P	Visit1_6	ERR2803754	Visit2_80	ERR2803935	Visit3_51	ERR2803986	Visit4_71	ERR2804088	Visit5_50	ERR2804143
52	T1	Visit1_7	ERR2803765	Visit2_81	ERR2803936	Visit3_52	ERR2803987	Visit4_72	ERR2804089	Visit5_51	ERR2804144
53	T2	Visit1_81	ERR2803767	Visit2_47	ERR2803898	Visit3_53	ERR2803988	Visit4_73	ERR2804090	Visit5_52	ERR2804145
54	P	Visit1_63	ERR2803747	Visit2_48	ERR2803899	Visit3_54	ERR2803989	Visit4_74	ERR2804091	Visit5_53	ERR2804146
55	T3	Visit1_65	ERR2803749	Visit2_49	ERR2803900	Visit3_55	ERR2803990	Visit4_75	ERR2804092	Visit5_54	ERR2804147
56	T1	Visit1_92	ERR2803779	Visit2_50	ERR2803902	Visit3_56	ERR2803991	Visit4_76	ERR2804093	Visit5_55	ERR2804148
57	P	Visit1_62	ERR2803746	Visit2_51	ERR2803903	Visit3_57	ERR2803992	NA	NA	NA	NA
58	T3	Visit1_89	ERR2803775	Visit2_52	ERR2803904	Visit3_58	ERR2803993	Visit4_77	ERR2804094	Visit5_56	ERR2804149
59	T1	Visit1_90	ERR2803777	Visit2_53	ERR2803905	Visit3_59	ERR2803994	Visit4_78	ERR2804095	Visit5_57	ERR2804150
60	T2	Visit1_11	ERR2803690	Visit2_78	ERR2803932	Visit3_60	ERR2803996	NA	NA	NA	NA
61	T1	Visit1_25	ERR2803705	Visit2_54	ERR2803906	Visit3_61	ERR2803997	Visit4_12	ERR2804023	Visit5_58	ERR2804151
62	P	Visit1_40	ERR2803722	Visit2_55	ERR2803907	Visit3_62	ERR2803998	Visit4_11	ERR2804022	Visit5_59	ERR2804152
63	T2	Visit1_42	ERR2803724	Visit2_56	ERR2803908	Visit3_63	ERR2803999	Visit4_10	ERR2804021	Visit5_60	ERR2804154

Subject	Dosage	Day1	Accession number	Day60	Accession number	Day75	Accession number	Day 90	Accession number	Day 120	Accession number
64	T3	Visit1_43	ERR2803725	Visit2_57	ERR2803909	Visit3_64	ERR2804000	Visit4_9	ERR2804098	Visit5_61	ERR2804155
65	T1	Visit1_45	ERR2803727	Visit2_58	ERR2803910	Visit3_65	ERR2804001	Visit4_8	ERR2804097	Visit5_62	ERR2804156
66	T2	Visit1_83	ERR2803769	Visit2_59	ERR2803911	Visit3_66	ERR2804002	Visit4_7	ERR2804096	Visit5_63	ERR2804157
67	T3	Visit1_82	ERR2803768	Visit2_60	ERR2803913	Visit3_67	ERR2804003	Visit4_6	ERR2804086	Visit5_64	ERR2804158
68	P	Visit1_85	ERR2803771	Visit2_61	ERR2803914	Visit3_68	ERR2804004	Visit4_5	ERR2804075	Visit5_65	ERR2804159
69	P	Visit1_84	ERR2803770	Visit2_62	ERR2803915	Visit3_69	ERR2804005	Visit4_4	ERR2804064	Visit5_66	ERR2804160
70	T2	Visit1_86	ERR2803772	Visit2_63	ERR2803916	Visit3_70	ERR2804007	Visit4_3	ERR2804053	Visit5_67	ERR2804161
71	T3	Visit1_24	ERR2803704	Visit2_64	ERR2803917	Visit3_71	ERR2804008	Visit4_2	ERR2804042	Visit5_68	ERR2804162
72	T1	Visit1_30	ERR2803711	Visit2_65	ERR2803918	Visit3_72	ERR2804009	Visit4_1	ERR2804031	Visit5_69	ERR2804163
73	P	Visit1_44	ERR2803726	Visit2_66	ERR2803919	Visit3_73	ERR2804010	Visit4_20	ERR2804032	Visit5_70	ERR2804165
74	T3	Visit1_31	ERR2803712	Visit2_67	ERR2803920	Visit3_74	ERR2804011	Visit4_19	ERR2804030	Visit5_71	ERR2804166
75	T2	Visit1_38	ERR2803719	Visit2_68	ERR2803921	Visit3_75	ERR2804012	Visit4_18	ERR2804029	Visit5_72	ERR2804167
76	T1	Visit1_29	ERR2803709	Visit2_69	ERR2803922	Visit3_76	ERR2804013	Visit4_17	ERR2804028	Visit5_73	ERR2804168
77	T2	Visit1_28	ERR2803708	Visit2_70	ERR2803924	Visit3_77	ERR2804014	Visit4_16	ERR2804027	Visit5_74	ERR2804169
78	T1	Visit1_34	ERR2803715	Visit2_83	ERR2803938	NA	NA	Visit4_15	ERR2804026	Visit5_75	ERR2804170
79	T3	Visit1_35	ERR2803716	Visit2_71	ERR2803925	Visit3_79	ERR2804016	Visit4_14	ERR2804025	Visit5_76	ERR2804171
80	P	Visit1_27	ERR2803707	Visit2_72	ERR2803926	Visit3_80	ERR2804018	Visit4_13	ERR2804024	Visit5_77	ERR2804172

Subject	Dosage	Day 150	Accession number	Day 165	Accession number	Day 180	Accession number	Day 210	Accession number
1	T1	Visit6_21	ERR2804188	Visit7_1	ERR2804262	Visit8_1	ERR2804338	Visit9_1	ERR2803791
2	P	Visit6_22	ERR2804189	Visit7_2	ERR2804273	Visit8_2	ERR2804349	Visit9_2	ERR2803802
3	T2	Visit6_23	ERR2804190	Visit7_3	ERR2804284	Visit8_3	ERR2804360	Visit9_3	ERR2803813
4	T3	Visit6_24	ERR2804191	Visit7_4	ERR2804295	Visit8_4	ERR2804371	NA	NA
5	T3	Visit6_25	ERR2804192	Visit7_5	ERR2804306	Visit8_5	ERR2804382	Visit9_5	ERR2803835
6	T2	Visit6_26	ERR2804193	Visit7_6	ERR2804317	Visit8_6	ERR2804393	Visit9_6	ERR2803846
7	T1	Visit6_27	ERR2804194	NA	NA	Visit8_7	ERR2804400	Visit9_7	ERR2803855
8	P	Visit6_28	ERR2804195	Visit7_7	ERR2804325	Visit8_8	ERR2804401	Visit9_8	ERR2803856
9	T2	Visit6_29	ERR2804196	Visit7_8	ERR2804326	Visit8_9	ERR2804402	Visit9_9	ERR2803857
10	P	Visit6_30	ERR2804198	Visit7_9	ERR2804327	Visit8_10	ERR2804328	Visit9_10	ERR2803781
11	T3	Visit6_31	ERR2804199	Visit7_10	ERR2804252	Visit8_11	ERR2804329	Visit9_11	ERR2803782
12	T1	Visit6_32	ERR2804200	Visit7_11	ERR2804253	Visit8_12	ERR2804330	Visit9_12	ERR2803783
13	T3	NA	NA	Visit7_12	ERR2804254	Visit8_13	ERR2804331	Visit9_13	ERR2803784

Subject	Dosage	Day 150	Accession number	Day 165	Accession number	Day 180	Accession number	Day 210	Accession number
14	T1	Visit6_33	ERR2804201	Visit7_13	ERR2804255	Visit8_14	ERR2804332	Visit9_14	ERR2803785
15	T2	Visit6_34	ERR2804202	Visit7_14	ERR2804256	NA	NA	NA	NA
16	P	Visit6_35	ERR2804203	Visit7_15	ERR2804257	NA	NA	NA	NA
17	T3	Visit6_36	ERR2804204	Visit7_16	ERR2804258	Visit8_16	ERR2804334	Visit9_17	ERR2803788
18	T1	Visit6_37	ERR2804205	Visit7_17	ERR2804259	NA	NA	Visit9_18	ERR2803789
19	P	Visit6_38	ERR2804206	Visit7_18	ERR2804260	Visit8_17	ERR2804335	Visit9_19	ERR2803790
20	T2	Visit6_39	ERR2804207	Visit7_19	ERR2804261	Visit8_18	ERR2804336	Visit9_20	ERR2803792
21	T1	NA	NA	NA	NA	NA	NA	NA	NA
22	T3	Visit6_40	ERR2804209	Visit7_20	ERR2804263	Visit8_19	ERR2804337	Visit9_21	ERR2803793
23	P	Visit6_41	ERR2804210	Visit7_21	ERR2804264	Visit8_20	ERR2804339	Visit9_22	ERR2803794
24	T2	Visit6_42	ERR2804211	Visit7_22	ERR2804265	Visit8_21	ERR2804340	Visit9_23	ERR2803795
25	T3	Visit6_43	ERR2804212	Visit7_23	ERR2804266	Visit8_22	ERR2804341	Visit9_24	ERR2803796
26	T2	Visit6_44	ERR2804213	Visit7_24	ERR2804267	Visit8_23	ERR2804342	Visit9_25	ERR2803797
27	T1	Visit6_45	ERR2804214	Visit7_25	ERR2804268	Visit8_24	ERR2804343	Visit9_26	ERR2803798
28	P	Visit6_46	ERR2804215	Visit7_26	ERR2804269	NA	NA	Visit9_27	ERR2803799
29	T2	Visit6_47	ERR2804216	Visit7_27	ERR2804270	Visit8_26	ERR2804345	Visit9_28	ERR2803800
30	T3	Visit6_48	ERR2804217	Visit7_28	ERR2804271	Visit8_27	ERR2804346	Visit9_29	ERR2803801
31	T1	Visit6_49	ERR2804218	Visit7_29	ERR2804272	Visit8_28	ERR2804347	Visit9_30	ERR2803803
32	P	Visit6_50	ERR2804220	Visit7_30	ERR2804274	Visit8_29	ERR2804348	Visit9_31	ERR2803804
33	T2	Visit6_51	ERR2804221	Visit7_31	ERR2804275	Visit8_30	ERR2804350	Visit9_32	ERR2803805
34	T3	Visit6_52	ERR2804222	Visit7_32	ERR2804276	Visit8_31	ERR2804351	Visit9_33	ERR2803806
35	P	Visit6_53	ERR2804223	Visit7_33	ERR2804277	Visit8_32	ERR2804352	Visit9_34	ERR2803807
36	T1	Visit6_54	ERR2804224	Visit7_34	ERR2804278	Visit8_33	ERR2804353	Visit9_35	ERR2803808
37	T3	Visit6_55	ERR2804225	Visit7_35	ERR2804279	Visit8_34	ERR2804354	Visit9_36	ERR2803809
38	P	Visit6_56	ERR2804226	Visit7_36	ERR2804280	Visit8_35	ERR2804355	Visit9_37	ERR2803810
39	T2	Visit6_57	ERR2804227	Visit7_37	ERR2804281	Visit8_36	ERR2804356	Visit9_38	ERR2803811
40	T1	Visit6_58	ERR2804228	Visit7_38	ERR2804282	Visit8_37	ERR2804357	Visit9_39	ERR2803812
41	T1	Visit6_59	ERR2804229	Visit7_39	ERR2804283	Visit8_38	ERR2804358	Visit9_40	ERR2803814
42	P	Visit6_60	ERR2804231	Visit7_40	ERR2804285	Visit8_39	ERR2804359	Visit9_41	ERR2803815
43	T3	Visit6_61	ERR2804232	Visit7_41	ERR2804286	Visit8_40	ERR2804361	Visit9_42	ERR2803816
44	T2	Visit6_62	ERR2804233	Visit7_42	ERR2804287	Visit8_41	ERR2804362	Visit9_43	ERR2803817
45	T1	Visit6_63	ERR2804234	Visit7_43	ERR2804288	Visit8_42	ERR2804363	Visit9_44	ERR2803818
46	T3	Visit6_64	ERR2804235	Visit7_44	ERR2804289	Visit8_43	ERR2804364	Visit9_45	ERR2803819

Subject	Dosage	Day 150	Accession number	Day 165	Accession number	Day 180	Accession number	Day 210	Accession number
47	T2	Visit6_65	ERR2804236	Visit7_45	ERR2804290	Visit8_44	ERR2804365	Visit9_46	ERR2803820
48	P	Visit6_66	ERR2804237	Visit7_46	ERR2804291	Visit8_45	ERR2804366	Visit9_47	ERR2803821
49	T2	Visit6_67	ERR2804238	Visit7_47	ERR2804292	Visit8_46	ERR2804367	Visit9_48	ERR2803822
50	T3	Visit6_68	ERR2804239	Visit7_48	ERR2804293	Visit8_47	ERR2804368	Visit9_49	ERR2803823
51	P	Visit6_69	ERR2804240	Visit7_49	ERR2804294	Visit8_48	ERR2804369	Visit9_50	ERR2803825
52	T1	Visit6_70	ERR2804242	Visit7_50	ERR2804296	Visit8_49	ERR2804370	Visit9_51	ERR2803826
53	T2	Visit6_71	ERR2804243	Visit7_51	ERR2804297	Visit8_50	ERR2804372	Visit9_52	ERR2803827
54	P	Visit6_72	ERR2804244	Visit7_52	ERR2804298	Visit8_51	ERR2804373	Visit9_53	ERR2803828
55	T3	Visit6_73	ERR2804245	Visit7_53	ERR2804299	Visit8_52	ERR2804374	Visit9_54	ERR2803829
56	T1	Visit6_74	ERR2804246	Visit7_54	ERR2804300	Visit8_53	ERR2804375	Visit9_55	ERR2803830
57	P	NA	NA	NA	NA	NA	NA	NA	NA
58	T3	Visit6_75	ERR2804247	Visit7_55	ERR2804301	Visit8_54	ERR2804376	Visit9_56	ERR2803831
59	T1	Visit6_76	ERR2804248	Visit7_56	ERR2804302	Visit8_55	ERR2804377	Visit9_57	ERR2803832
60	T2	NA	NA	NA	NA	NA	NA	NA	NA
61	T1	Visit6_12	ERR2804178	Visit7_57	ERR2804303	Visit8_56	ERR2804378	Visit9_58	ERR2803833
62	P	Visit6_11	ERR2804177	Visit7_58	ERR2804304	Visit8_57	ERR2804379	Visit9_59	ERR2803834
63	T2	Visit6_10	ERR2804176	Visit7_59	ERR2804305	Visit8_58	ERR2804380	Visit9_60	ERR2803836
64	T3	Visit6_9	ERR2804251	Visit7_60	ERR2804307	Visit8_59	ERR2804381	Visit9_61	ERR2803837
65	T1	Visit6_8	ERR2804250	Visit7_61	ERR2804308	Visit8_60	ERR2804383	Visit9_62	ERR2803838
66	T2	Visit6_7	ERR2804249	Visit7_62	ERR2804309	Visit8_61	ERR2804384	Visit9_63	ERR2803839
67	T3	Visit6_6	ERR2804241	Visit7_63	ERR2804310	Visit8_62	ERR2804385	Visit9_64	ERR2803840
68	P	Visit6_5	ERR2804230	Visit7_64	ERR2804311	Visit8_63	ERR2804386	Visit9_65	ERR2803841
69	P	Visit6_4	ERR2804219	Visit7_65	ERR2804312	Visit8_64	ERR2804387	Visit9_66	ERR2803842
70	T2	Visit6_3	ERR2804208	Visit7_66	ERR2804313	Visit8_65	ERR2804388	Visit9_67	ERR2803843
71	T3	Visit6_2	ERR2804197	Visit7_67	ERR2804314	Visit8_66	ERR2804389	Visit9_68	ERR2803844
72	T1	Visit6_1	ERR2804186	Visit7_68	ERR2804315	Visit8_67	ERR2804390	Visit9_69	ERR2803845
73	P	Visit6_20	ERR2804187	Visit7_69	ERR2804316	Visit8_68	ERR2804391	Visit9_70	ERR2803847
74	T3	Visit6_19	ERR2804185	Visit7_70	ERR2804318	Visit8_69	ERR2804392	Visit9_71	ERR2803848
75	T2	Visit6_18	ERR2804184	Visit7_71	ERR2804319	Visit8_70	ERR2804394	Visit9_72	ERR2803849
76	T1	Visit6_17	ERR2804183	Visit7_72	ERR2804320	Visit8_71	ERR2804395	Visit9_73	ERR2803850
77	T2	Visit6_16	ERR2804182	Visit7_73	ERR2804321	Visit8_72	ERR2804396	Visit9_74	ERR2803851
78	T1	Visit6_15	ERR2804181	Visit7_74	ERR2804322	Visit8_73	ERR2804397	Visit9_75	ERR2803852
79	T3	Visit6_14	ERR2804180	Visit7_75	ERR2804323	Visit8_74	ERR2804398	Visit9_76	ERR2803853
80	P	Visit6_13	ERR2804179	Visit7_76	ERR2804324	Visit8_75	ERR2804399	Visit9_77	ERR2803854
